# Exploration of differential expression and biological significance of amino acid metabolism genes in osteoarthritis

**DOI:** 10.3389/fimmu.2025.1588072

**Published:** 2025-07-14

**Authors:** Zhenghuan Zhu, Jiaqing Meng, Junfeng Hu, Lingmin Hu, Wenge Ding, Wanchao Zhang, Chuang Zhao, Lin Feng, Kejie Wang

**Affiliations:** ^1^ Department of Orthopedics, Changzhou First People’s Hospital, Third Affiliated Hospital of Soochow University, Changzhou, Jiangsu, China; ^2^ Department of Reproduction, Changzhou Maternity and Child Health Care Hospital, Changzhou, Jiangsu, China; ^3^ Department of Surgery, Wuqia County People’s Hospital, Uqia, Xinjiang, China

**Keywords:** osteoarthritis, amino acid metabolism genes, weighted gene co-expression network analysis, immunity-related genes, immune infiltration, lasso regression, SVM-RFE, single-cell analysis

## Abstract

**Background:**

Osteoarthritis (OA) is a widespread disorder affecting joints, recognized for cartilage wear and inflammatory responses, which substantially affects patients’ quality of life. This research aim to discover amino acid metabolism-related differentially expressed genes (AAMRDEGs) and clarify their functions in OA pathogenesis.

**Methods:**

Herein, we conducted an analysis of combined GEO datasets (GSE55457, GSE55235, and GSE12021), identifying 169 AAMRDEGs and indicating their importance in chondrocyte function and inflammation. Furthermore, significant correlations were observed between various immune cell types, underscoring the intricate function of the immune system in OA. Thereafter, we developed highly accurate diagnostic models using LASSO regression and SVM methodologies, achieving an area under the curve > 0.9. Protein-protein interaction analysis revealed significant interactions among MTHFD2, PPP1R15A, SLC2A4, and WNT5B, with their expression levels corroborated using single-cell datasets, highlighting the potential therapeutic targets. To confirm the presence of these hub AAMRGs, real-time polymerase chain reaction and immunohistochemistry were employed.

**Results:**

We identified 2,115 DEGs between OA and control groups, with 1,062 upregulated and 1,053 downregulated. Enrichment analysis linked AAMRDEGs to amino acid catabolism and multiple KEGG pathways, indicating their importance in chondrocyte function and inflammation. Furthermore, significant correlations were observed between various immune cell types, underscoring the intricate role of the immune system in OA. Subsequently, we developed highly accurate diagnostic models using LASSO regression and SVM methodologies, achieving an area under the curve > 0.9. Protein-protein interaction analysis revealed significant interactions among *MTHFD2, PPP1R15A, SLC2A4*, and *WNT5B*, with their expression levels corroborated using single-cell datasets, highlighting the potential therapeutic targets. Real-time polymerase chain reaction and immunohistochemistry were used to validate the expression of these hub amino acid metabolism-related genes.

**Conclusion:**

This investigation presents a detailed evaluation of AAMRGs in OA, highlighting their roles in disease pathogenesis and offering new insights for therapeutic research. Key genes *SLC2A4, MTHDF2*, and *WNT5B* might function as markers for early identification and personalized OA treatment.

## 1 Introduction

Osteoarthritis (OA) is a widespread disorder affecting joints that markedly diminishes the standard of living for millions worldwide, resulting in a considerable socioeconomic burden. In China, there was a notable rise of prevalent incident cases and years lived with disability (YLDs) associated with OA, growing significantly from 51.8, 4.6, and 1.8 million, in 1990, to 132.8, 10.7, and 4.7 million, respectively, in 2019 (1, 2). Despite its high prevalence, efficacious therapeutic strategies are limited, predominantly focusing on symptomatic management through the use of analgesics, anti-inflammatory drugs, physical therapy, and, in extreme cases, surgical options such as joint replacement (3). However, these approaches frequently provide insufficient relief and are accompanied by adverse side effects, underscoring the pressing necessity for innovative targeted therapeutics to tackle OA fundamental pathophysiological mechanisms.

Periarticular structures, including the synovium and cartilage, are integral to OA pathogenesis. The synovium is responsible for the production of synovial fluid, which lubricates the joint and nourishes the cartilage. Inflammation of the synovial membrane is a prominent feature of OA, marked by immune cell infiltration (ICI) and the release of proinflammatory cytokines, further exacerbating cartilage degradation and contributing to pain (4). Conversely, cartilage is vital for joint function, and its degeneration induces the hallmark symptoms of OA. The interaction between synovial inflammation and cartilage degradation highlights the necessity of addressing both structures in therapeutic strategies to slow OA progression. Furthermore, due to the relative accessibility of periarticular tissues, the use of multi-omics technologies in research is on the rise. These studies investigated the pathological mechanisms underlying OA by analyzing the interactions between various biological layers, including RNA, proteins, and metabolites (5, 6). This approach improves our understanding of the complexity and dynamics of biological networks.

Recent developments in metabolomics have elucidated the impact of metabolic alterations in OA pathogenesis (7, 8). Emerging research has highlighted that disruption of amino acid metabolism (AAM) is a critical pathophysiological mechanism in arthritis. These metabolic pathways offer promising opportunities as diagnostic markers and therapeutic targets for OA (9). Substantial alterations in the amino acid profile have been documented in both local and systemic osteoarthritic joints, as evidenced by animal models and human studies. These alterations are intricately associated with the inflammatory state of the disease, cartilage degradation, and clinical manifestations (10). Fluctuating levels of metabolites, including branched-chain amino acids (BCAAs), arginine, and alanine, have been implicated in disease progression, offering potential avenues for the identification of novel diagnostic markers and therapeutic strategies. For instance, a recent study employed bioinformatics and machine learning methodologies to identify BCAA-related genes, such as SLC3A2 and SLC7A5, as prospective diagnostic markers for OA (11). Animal models are indispensable in elucidating the causal relationship between amino acid metabolism disorders and the pathogenesis of OA. They also serve to validate novel metabolism-related therapeutic intervention strategies and enhance the dynamic understanding of the disease’s pathological processes. Concurrently, multi-omics analyses of human clinical samples have advanced the translation of findings from animal model research into clinical practice, offering both a theoretical foundation and empirical evidence for the personalized diagnosis and treatment of OA. Despite these insights, the specific mechanisms by which AAM influences OA progression remain unexplored, indicating a significant gap in current knowledge.

In conjunction with metabolic disturbances, epigenetic and post-transcriptional regulation are critical factors in OA. Notably, miRNAs have been documented to affect inflammation, cartilage degradation, and immune cell activity. Incorporating miRNA analysis into transcriptomic studies facilitates the elucidation of upstream regulatory networks that may contribute to metabolic and immune dysfunction in OA (10).

This investigation aimed to clarify the principal genes and pathways linked to OA pathophysiology using an extensive array of bioinformatics techniques, with a specific focus on AAM-related genes (AAMRGs). This approach facilitates the identification of critical molecular components and their interactions within the context of OA, thereby offering potential avenues for identifying novel diagnostic biomarkers and therapeutic targets.

## 2 Materials and methods

### 2.1 Data acquisition

The OA datasets GSE55457, GSE55235, and GSE12021 (12, 13) were downloaded from the GEO database utilizing the R package GEO query (Version 2.70.0) (14). Every sample within these datasets was derived from *Homo sapiens*, with synovial membrane tissue as the tissue source (**Supplementary Table S1**). A comprehensive search in the GeneCards database yielded 996 AAMRGs. Furthermore, the relevant literature was extensively reviewed in the PubMed database (15), yielding a final compilation of 1204 AAMRGs (**Supplementary Table S2**). The combined dataset was derived by de-batching the GSE55457, GSE55235, and GSE12021 datasets using the R package sva (version 3.50.0) (16). This combined dataset comprised 29 OA and 29 control samples. Subsequently, normalization was applied to the datasets via the R package limma (version 3.58.1) (17), including probe annotation and additional standardization procedures. Principal component analysis (PCA) was conducted on the expression matrix prior to and following batch-effect removal to evaluate the effectiveness of the de-batching process.

### 2.2 Delineation of AAMRDEGs of OA

The samples were classified into OA and control groups depending on the categorization of the combined datasets. Using the limma, we investigated the differential gene expression. The criteria of |logFC| > 0.5 and *p* < 0.05 were established to identify differentially expressed genes (DEGs). The Benjamini-Hochberg procedure was applied to correct *p*-values. The outcomes of the differential analysis were presented in volcano plots created via the ggplot2 R software (version 3.4.4). In order to find OA-related AAMRDEGs, all DEGs that were obtained from the combined datasets’ differential analysis and had a |logFC| > 0.5 and a *p*-value < 0.05 were intersected with AAMRGs. This intersection was represented by a Venn diagram. Subsequently, the AAMRDEGs were identified, and a heatmap illustrating the top 20 AAMRDEGs was visualized in a heatmap using pheatmap (Version 1.0.12).

### 2.3 Gene ontology and Kyoto Encyclopedia of Genes and Genomes enrichment analyses of AAMRDEGs

Both GO and KEGG analyses were carried out to clarify the biological significance of DEGs and the associated pathways (18, 19). The impact of AAMRGs on biological processes (BP), molecular functions (MF), and cellular components (CC) was comprehensively analyzed via the R package clusterProfiler (version 4.10.0) (20). This study aimed to outline the main biological themes and molecular pathways affected by these genes, which could improve our comprehension of their roles in OA pathology and help identify possible therapeutic targets. The item screening criteria were *p* < 0.05 and false discovery rate value < 0.05; the Benjamini–Hochberg method was employed for *p*-value correction.

### 2.4 Gene set enrichment analysis and gene set variation analysis

Both GSEA (21) and GSVA (22) were employed to detect functional gene sets and pathway alterations across different samples in the combined datasets comparing OA and control groups. These analyses, performed using R, highlighted the active BP and pathways across various risk groups by examining AAMRDEGs and their impact on BP, MF, CC, and pathways, thereby giving in-depth explanations of disease mechanisms. Both GSVA and GSEA analyses yielded *p* < 0.05, with the Benjamini–Hochberg method employed for *p*-value correction.

### 2.5 Weighted correlation network analysis

The integrated GEO dataset related to OA was assessed via the R package WGCNA (23). Correlation coefficients between DEGs with |logFC| > 0 and *p* < 0.05 were calculated to ensure the construction of a scale-free network. A hierarchical clustering tree was then constructed to identify gene modules, with parameters set to a minimum module size of 50 genes and a soft-thresholding power of 8. Modules were selected based on their correlation values, and all genes within these modules were identified as DEGs that were significantly associated with OA.

### 2.6 Construction of OA diagnostic model

To construct a GEO dataset for the OA diagnostic model, AAMRDEGs were evaluated using logistic regression, with a binary dependent variable distinguishing OA from control. A significance threshold of *p* < 0.05 was employed to filter AAMRDEGs for inclusion in the model. To mitigate overfitting, LASSO regression with a penalty term was performed using the glmnet package (24), incorporating a penalty term. The results were visualized using diagnostic plots. The AAMRDEGs were identified as model genes for the OA diagnostic model using LASSO analysis. The LASSO risk score was derived from the risk coefficients, and an SVM model (25) was developed using these AAMRDEGs, with the maximum accuracy and minimum error rate in gene selection.

### 2.7 Validation of OA diagnostic model

The R package ‘rms’ (Version 6.7-1) was utilized to create a nomogram for key genes, thereby elucidating the correlation between independent variables and risk within a regression model framework. By comparing the actual and expected probabilities through a calibration plot, with a focus on logistic regression, we were able to evaluate the model’s prediction accuracy. The clinical value of the prediction models was estimated by decision curve analysis, and the accuracy of the logistic regression model was established via the ‘ggDCA’ software (Version 1.1). In order to estimate the regression model’s diagnostic performance for OA, the ‘pROC’ package (Version 1.18.5) was employed to generate receiver operating characteristic (ROC) curves and calculate the area under the curve (AUC) values from combined datasets. Better performance was indicated by closer AUC values, which ranged from 0.5 to 1.

### 2.8 Construction of protein-protein interaction network

The PPI network includes distinct proteins that interact with each other. To identify known and predicted protein interactions, the STRING database was used. This investigation utilized the STRING database (26) to create a PPI network centered on key genes, specifying humans as the biological species, with a confidence threshold of ≥ 0.150 and limiting the number of interactors to a maximum of five. The constructed PPI network model was visualized via Cytoscape (27). Additionally, the GeneMANIA platform (28) was utilized for the prediction of functionally analogous genes associated with the identified key genes, thereby constructing an interaction network based on these predictions.

### 2.9 Construction of RNA regulatory network

To discover the interplay between key genes and miRNAs, the TarBase database was utilized to identify key genes, and Cytoscape software was utilized for visualizing the mRNA-miRNA regulatory network, exploring the interplay between these genes and miRNAs. Furthermore, transcription factors (TFs) modulate gene expression by engaging with essential genes in the post-transcriptional phase. Moreover, we retrieved relevant TFs from ChIPBase and examined the regulatory functions they play in important genes. The use of Cytoscape allowed the visualization of the mRNA-TF control network.

### 2.10 Differential expression analysis of OA Key genes

The Mann–Whitney U test was performed to investigate expression variations of key genes in combined OA and control datasets, aiming to clarify the mechanisms, biological characteristics, and pathways linked to DEGs in OA. Afterward, ggplot2 comparative mapping was used to illustrate the variance analysis results.

The variance analysis outcomes were quantitatively assessed based on key gene expression levels. This facilitated identifying the key genes in the combined dataset, which were subsequently analyzed using ROC curve analysis for improved result visualization. The ROC curve is a graphical representation tool that aids in choosing the optimal model, discarding suboptimal alternatives, or determining the best threshold values within a given model (29). This curve integrates the sensitivity and specificity of the continuous variables, thereby illustrating their interrelationships. The R programming package was employed to construct ROC curves following the screening of key genes within the integrated dataset. The diagnostic effectiveness of key gene expression levels in OA was evaluated using the AUC calculation.

### 2.11 Immune infiltration analysis

To measure the relative abundance of numerous ICI, single-sample GSEA (ssGSEA) (30) was used. Initially, various infiltrating immune cells were identified, including but not limited to activated CD8^+^T cells, gamma-delta T cells, natural killer cells, and subtypes like regulatory T cells (Tregs). Subsequently, the ssGSEA enrichment scores quantified the relative abundance of each immune cell type across samples, resulting in an ICI matrix for the combined GEO datasets. The R package ggplot2 was utilized to create comparative graphical representations, highlighting differences in immune cell expression across various groups within the datasets.

Immune cells showing significant variations between both groups were chosen for further study. The Spearman algorithm was used to determine correlations among immune cells. The R package pheatmap was then utilized to produce heat maps that illustrate the correlation analysis outcomes among the immune cells. Besides, based on the Spearman correlation, correlation bubble charts were created by the ggplot2 package to depict relationships between key genes and immune cells.

### 2.12 Construction of OA subtypes

Consistency Clustering, which is based on a resampling algorithm, is used to determine the membership of each individual within its respective group and evaluate the validity of the clustering process (31). This method, known as consensus clustering, involves multiple iterations of dataset subsamples, thereby offering a measure of cluster stability and aiding parameter selection by introducing sampling variability. Using the R package Consensus Cluster Plus, the consensus clustering technique focused on key genes to identify combined datasets of OA samples across various disease subtypes. In this analysis, the count of clusters was predetermined to a range of 2-9, with the process being repeated 50 times, extracting 80% of the total sample each time. The clustering algorithm was “km,” and the distance metric used was “Pearson.” Subsequently, heatmap analysis was performed to estimate the key genes’ expression levels within the combined OA datasets, highlighting the expression differences among samples from different disease subtypes. The validation of key genes was further conducted through comparative expression analysis across disease subtypes.

### 2.13 Quality control of single-cell dataset

The GSE152805 dataset (32) from Homo sapiens, sourced from synovial tissue via GPL20301, included three OA cases and six controls. A Seurat object was created from the count matrix by applying additional filters, like Count < 500 and logGenesPerUMI < 0.8, to identify cells with at least three genes and 200 expressions. The scRNA-seq data was normalized via the ‘LogNormalize’ method, followed by identifying the top 2000 variable genes with ‘Find Variable Features’ and subsequent data scaling. The “ScaleData” function was used to mitigate the impact of sequencing depth. PCA was performed to detect substantial principal components (PCs), and the ‘ElbowPlot’ function was utilized to visualize *p*-value distribution. A total of 30 PCs were chosen for further analysis using Uniform Manifold Approximation and Projection (UMAP) to accomplish dimensionality reduction. A PCA-based K-nearest neighbor graph was generated in the Euclidean space using the default parameters of the “FindNeighbors” function, incorporating the 30 PCs. The “FindClusters” and the “clustree” functions were then used to determine that a resolution of 0.6 allowed for the division of cells into distinct clusters. To explore and visualize datasets, the “RunUMAP” function made dimensionality reduction easier.

### 2.14 Cell type annotation and single-cell taxa differential genes

Cell type annotation and identification were performed using cell marker genes in the single-cell GSE152805 dataset. Subsequently, the “DotPlot” and “FeaturePlot” functions were utilized to illustrate the key genes’ expression levels across different cell types. To identify variations in gene expression across clusters of cells, the “FindAllMarkers” function was utilized along with the Wilcoxon rank-sum test. Eventually, we chose the top ten DEGs for each cell cluster to serve as representative genes for single-cell populations in further studies.

### 2.15 AUCell analysis

AUCell is a tool designed to detect cells with active gene sets in single-cell RNA-sequencing data (33). The AUC metric assesses if a notable portion of the specified gene set is enriched among the expressed genes in individual cells. The analysis of AUC score distribution facilitated the evaluation of expression properties in all cells. Given that the scoring methodology relies on a ranking system, AUCell operates based on gene expression units and the effects of standardized protocols. Furthermore, since cells are evaluated on an individual basis, this approach is readily applicable to larger datasets and can accommodate expression matrices organized as required. In this study, we selected the top 10 DEGs for AUCell scoring and subsequently identified the cell populations exhibiting elevated scores.

### 2.16 Quantitative real-time PCR

RNA was extracted with a TRIzol reagent (Thermo Fisher, USA). Complementary DNA synthesis was conducted via PrimeScript™ RT Master Mix (Takara, Japan) per protocols. The qRT-PCR was conducted on the QuantStudio 5 system (Thermo Fisher, USA) utilizing iTaq™ Universal SYBR Green Supermix (Bio-Rad Laboratories, USA). The thermal cycling protocol began with a 3-min denaturation at 95°C, then 40 cycles of denaturation were carried out for 10 s and annealing/extension for 30 s at 60°C. Using internal controls, gene expression was standardized, and the 2^−ΔΔCT^ technique was used to quantify relative mRNA levels.

### 2.17 Immunohistochemical staining

Paraffin-embedded synovial tissue sections were subjected to dewaxing using three cycles of dewaxing solution, each lasting 10 min. This was followed by three washes in anhydrous ethanol, each for 5 min, and a subsequent rinse with distilled water. Antigen retrieval was conducted as specified, with careful maintenance of moisture in the buffer. Upon natural cooling, the sections underwent washes with phosphate-buffered saline (PBS) for 5 min. To prevent endogenous peroxidase activity, incubation of sections was carried out with 3% hydrogen peroxide for 25 min at room temperature. After that, they were washed with PBS. For 30 min at room temperature, tissue sections were treated with 3% bovine serum albumin (BSA). When primary antibodies originating from goats were used, rabbit serum was used for blocking. We incubated the sections overnight at 4°C with the primary antibody in PBS after removing the blocking solution. The slices were washed with PBS and then treated with a secondary antibody coated with horseradish peroxidase (HRP) for 50 min at room temperature. Diaminobenzidine (DAB) was used to achieve color development. The reaction was watched under a microscope, and when brown-yellow staining appeared, the reaction was terminated by washing with tap water. Finally, nuclear counterstaining was performed with hematoxylin for 3 min, followed by brief differentiation and rinsing with tap water until the nuclei returned to a blue hue.

### 2.18 Statistical analysis

The data analyses were conducted using the R software. Statistical significance for normally distributed continuous variables between two groups was assessed using the independent Student’s t-test unless otherwise specified. The Mann–Whitney U test was used to evaluate differences in non-normally distributed variables. The Kruskal–Wallis test was deployed to compare multiple groups. To evaluate the correlation between various molecules, Spearman correlation analysis was used to calculate the correlation coefficient. All statistical *p*-values were two-tailed unless indicated otherwise, with *p* < 0.05 denoting significance.

## 3 Results

### 3.1 Data collection and correction

**Figure 1:**
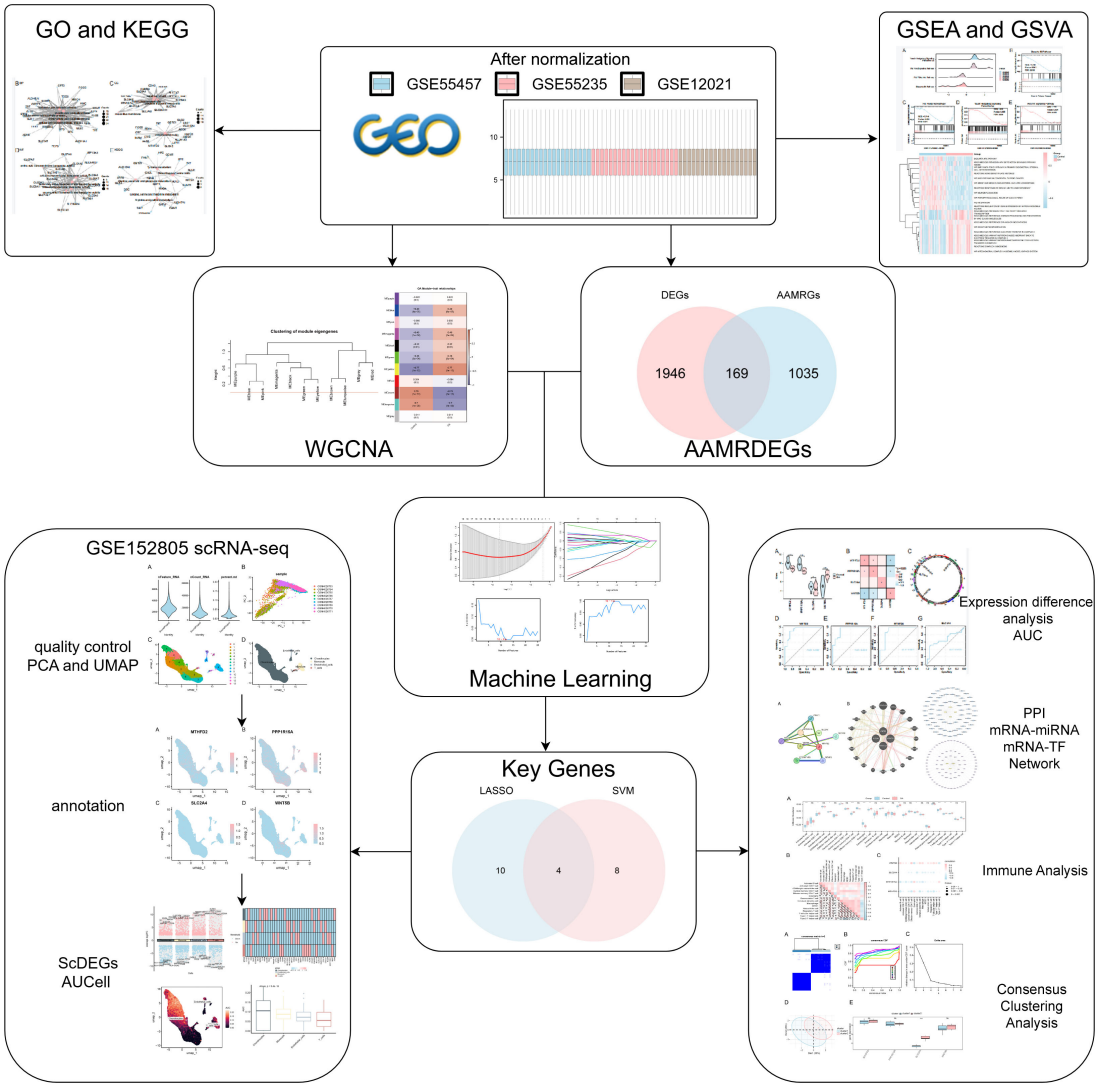
Diagram illustrating the analysis process flow.

To achieve consistency and comparability across multiple datasets, our initial objective was to integrate and normalize the original GEO datasets by eliminating batch effects. The R package sva was employed to address batch effects in OA datasets GSE55457, GSE55235, and GSE12021, resulting in the generation of combined GEO datasets. With the use of distribution boxplots, we compared the dataset expression values both before and after batch effect removal (**Supplementary Figures S1A, B**). Before and after batch effect correction, the distribution of low-dimensional features was evaluated using PCA plots (**Supplementary Figures S1C, D**). These findings show that the batch effect elimination procedure successfully removed batch effects from OA datasets. **Figure 1** displayed the central search.

### 3.2 OA-associated AAMRDEGs

**Figure 2:**
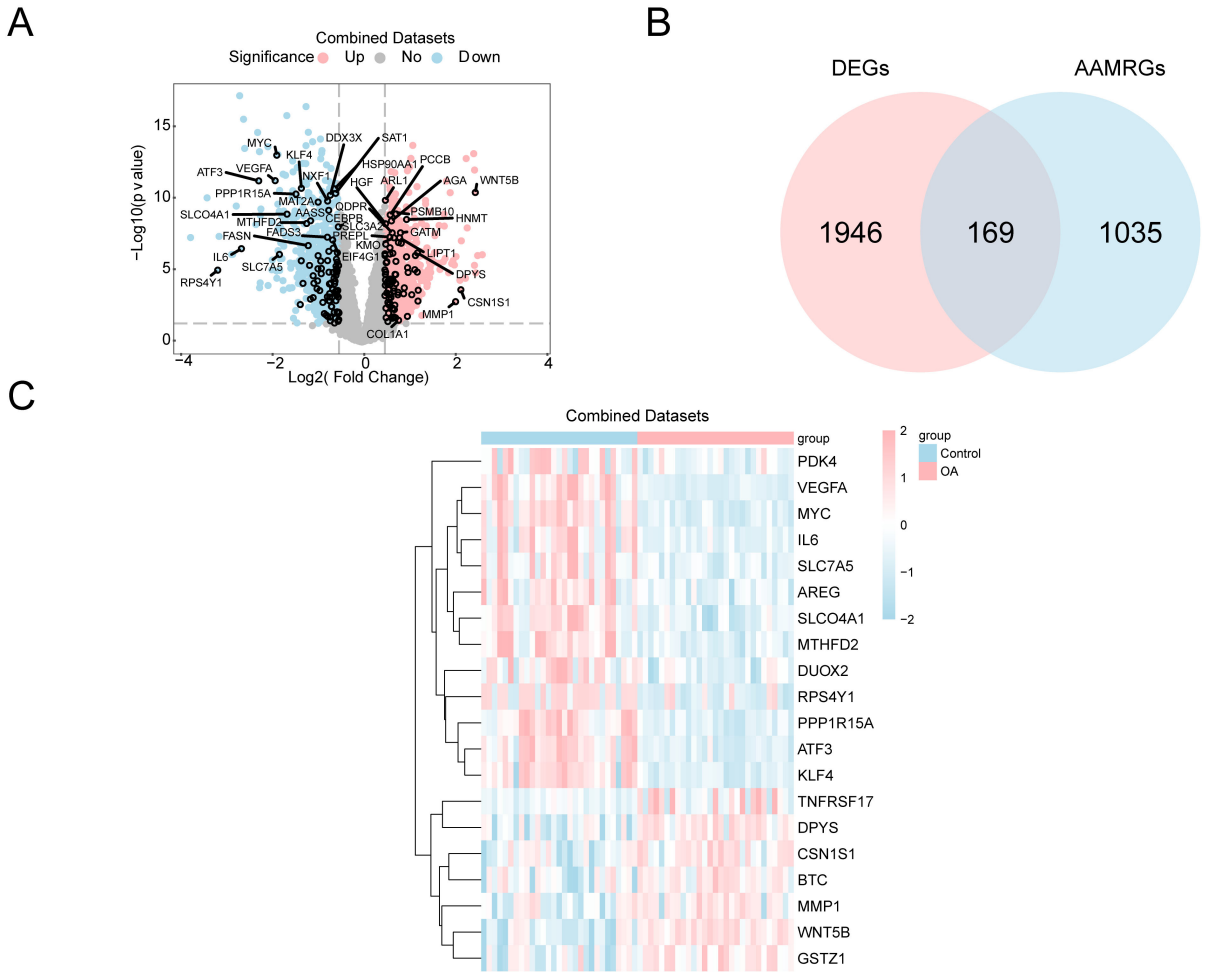
Differential gene expression analysis. **(A)** Volcano plot: DEG analysis between OA and control groups in the combined GEO datasets. **(B)** Venn diagram: DEGs and AAMRGs in the combined datasets. **(C)** Heatmap of AAMRDEGs in the combined datasets.

Differential expression analysis was conducted to explore OA-related transcriptomic changes, followed by intersection with amino acid metabolism-related genes to identify AAMRDEGs. There were two groups created from the combined GEO datasets: the OA and control groups. The limma was used for differential analysis to delve into the variations in gene expression between the groups. Among the 2,115 DEGs that fulfilled the conditions of |logFC| > 0.5 and *p* < 0.05, 1,062 genes were found to be upregulated and 1,053 genes to be downregulated (logFC < −0.5 and *p* < 0.05). The dataset’s differential analysis findings were used to construct a volcano plot (**Figure 2A**). Moreover, we built a Venn diagram (**Figure 2B**) to identify 169 AAMRDEGs by intersecting all DEGs with AAMRGs that had |logFC| > 0.5 and *p* < 0.05. Our analysis of the AAMRDEGs in the integrated GEO dataset across different sample groups was based on the intersection results. The top 20 AAMRDEGs’ expression patterns were visualized using a heatmap that was built using the pheatmap (**Figure 2C**).

### 3.3 Enrichment analysis of AAMRDEGs

**Figure 3:**
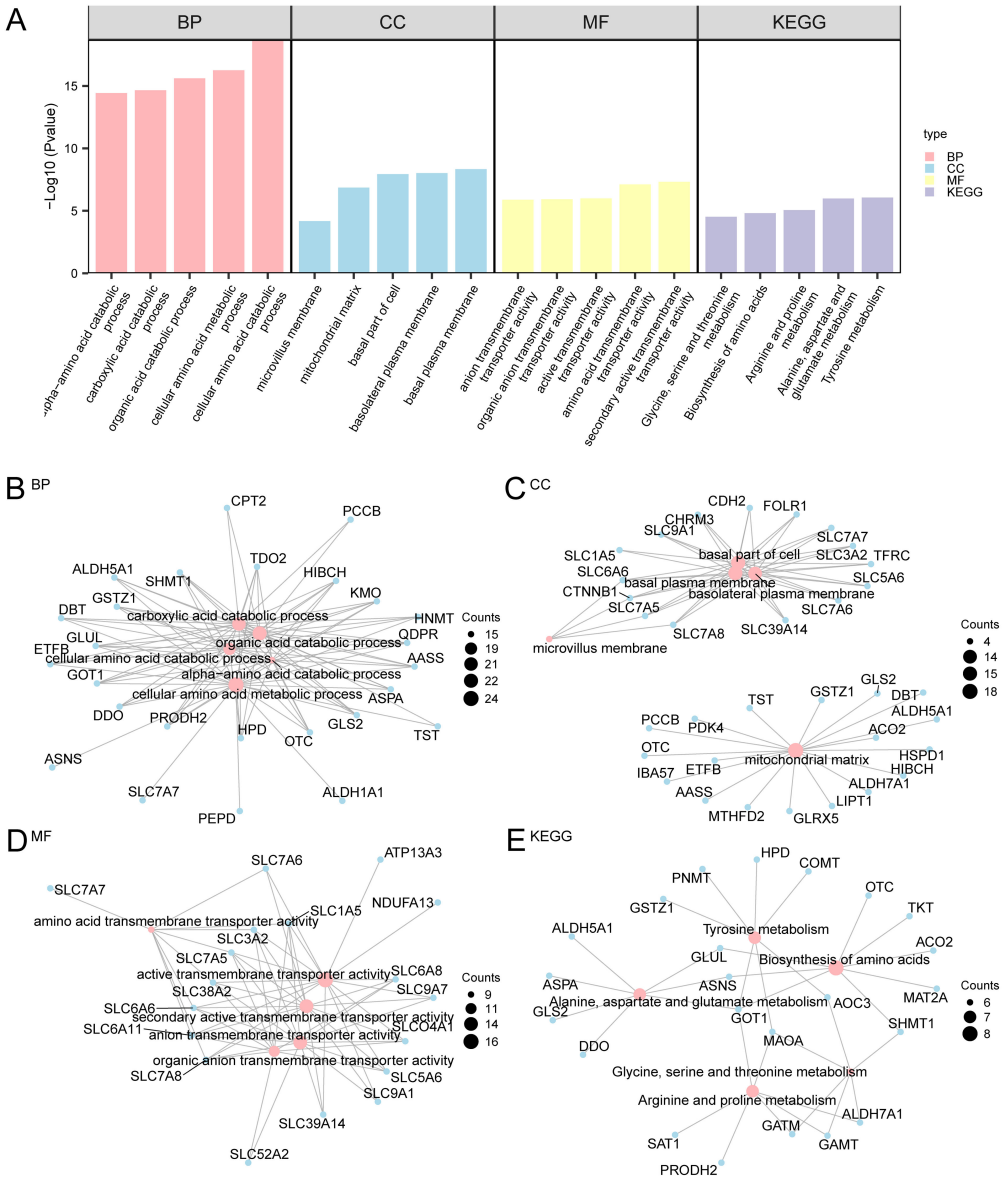
GO and KEGG enrichment analyses for AAMRDEGs. **(A)** Bar graph of GO and KEGG enrichment analysis results of AAMRDEGs: BP, CC, MF, and biological pathway. GO and KEGG terms are listed on the abscissa. **(B–E)** GO and KEGG results of AAMRDEGs: **(B)** BP, **(C)** CC, **(D)** MF and **(E)** KEGG. Pink nodes represent items, blue nodes represent molecules, and lines represent the relationship between items and molecules. The screening criteria for GO and KEGG enrichment analyses were *p* < 0.05 and FDR < 0.05. Benjamini-Hochberg method was used for *p* correction.

We next performed GO and KEGG enrichment analyses to investigate the functional implications of the identified AAMRDEGs in OA progression. A Gene Ontology enrichment analysis of the 169 differentially expressed genes associated with amino acid metabolism in OA identified significant associations across the three GO categories: biological process (BP), molecular function (MF), and cellular component (CC). Within the MF category, the genes were predominantly implicated in secondary active transporter activity and amino acid transmembrane transporter activity. The CC category demonstrated enrichment in the basal plasma membrane and mitochondrial matrix. In the BP category, the genes were primarily linked to cellular amino acid catabolic and metabolic processes. Furthermore, analysis using the KEGG pathway revealed a predominant involvement of these genes in tyrosine metabolism (**Figure 3A**). **Figures 3B–E** illustrate network diagrams for the BP, CC, MF, and KEGG pathways, where the size of each node reflects the number of genes associated with each term, and the connecting lines denote the relationships between genes and their respective annotations.

### 3.4 GSEA and GSVA in combined GEO datasets

To achieve a more thorough comprehension of pathway-level alterations associated with OA, we employed GSEA and GSVA to identify enriched biological processes and signaling pathways within the integrated dataset. The GSEA was employed to evaluate the effect of gene expression in the merged datasets on OA pathogenesis. The logFC values of all genes in the merged datasets were the basis of this analysis to compare between OA and control groups. The GSEA was utilized to determine the associations between gene expression and the corresponding BP, CC, and MF, and a mountain plot was used to visualize these associations (**Supplementary Figure S2A**). **Supplementary Table S3** displays the detailed findings. The findings demonstrated that a significant enrichment was found in the genes of the combined datasets in the IL6 (**Supplementary Figure S2B**), the PI3KCI AKT (**Supplementary Figure S2C**), and the Hedgehog signaling paracrine up pathways (**Supplementary Figure S2D**). Additionally, other biologically significant pathways and activities were enhanced, including Wnt signaling (**Supplementary Figure S2E**).

All genes in the integrated GEO dataset were subjected to GSVA in order to examine any variations in the c2.cp.v2023.2.Hs.symbols.gmt gene set between the OA and control groups (**Supplementary Table S4**). Afterward, the top 20 pathways (*p* < 0.05) were chosen and arranged in descending order by logFC absolute value. A heatmap was used to assess and illustrate the differential expression of the top 20 pathways between the OA and control groups (**Supplementary Figure S3A** **).** A comparative group diagram was used to show the data (**Supplementary Figure S3B**), and the Mann-Whitney U test was employed to examine the variations. Between the OA and control groups, there were statistically significant variations in pathways, including the Mitochondrial Complex I Assembly Model OXPHOS System and Complex I Biogenesis, according to the GSVA results (*p* < 0.05).

### 3.5 WGCNA analysis identified co-expression modules in the dataset

**Figure 4:**
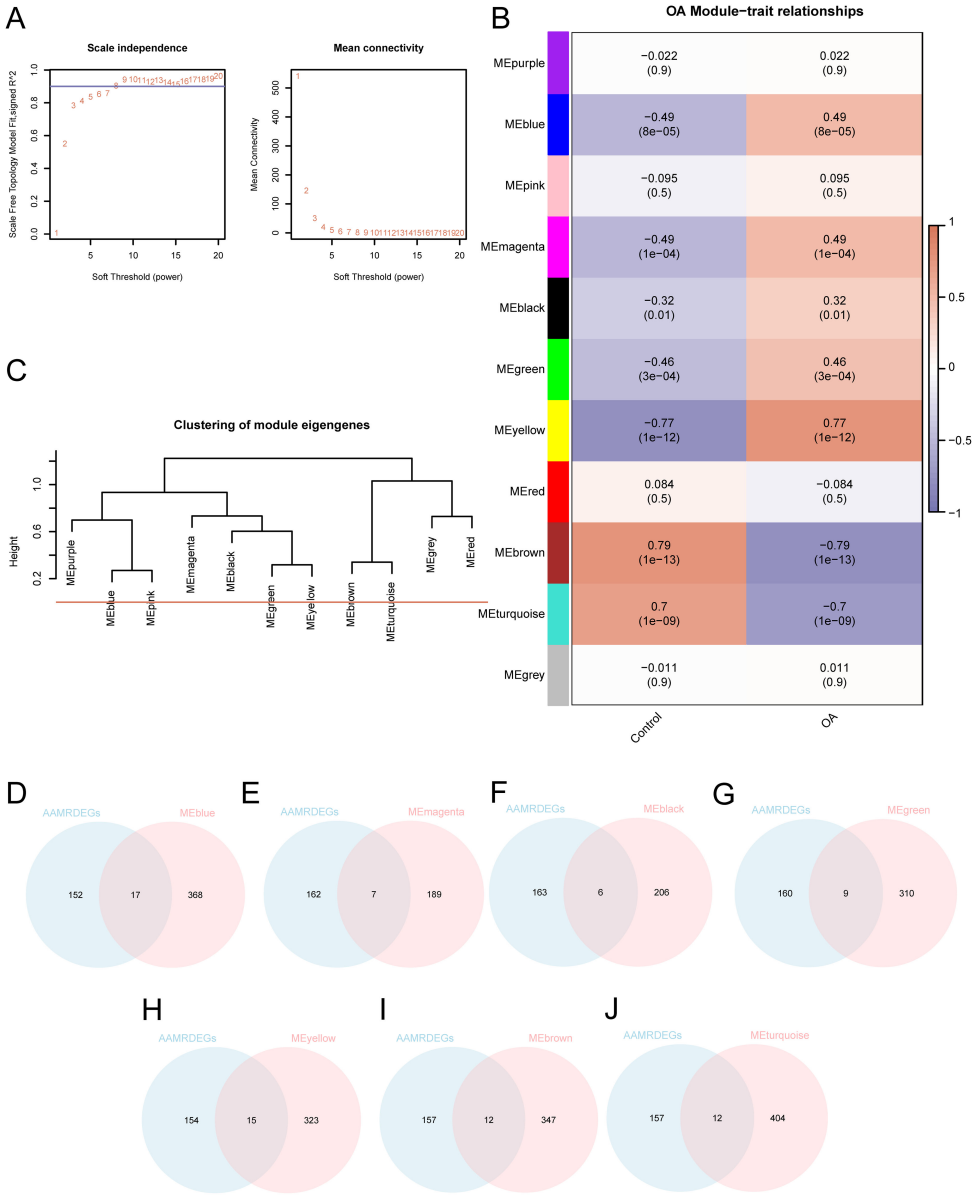
WGCNA analysis identified co-expression modules in the dataset. **(A)** Sample modules for OA combined datasets screening threshold scale-free network presentation. **(B)** OA integration of combined dataset DEGs module clustering and correlation analysis between different grouping results. **(C)** DEG module clustering results in OA-integrated GEO datasets (combined datasets). **(D–J)** OA integration of GEO dataset (combined datasets) of DEGs with **(D)** MEblue, **(E)** MEmagenta, **(F)** MEblack, **(G)** MEgreen, **(H)** MEyellow, **(I)** MEbrown, and **(J)** Venn diagram of MEturquoise.

A weighted gene co-expression network was constructed to identify OA-related gene modules and reveal potential co-regulatory patterns among AAMRDEGs. In order to find co-expression modules, we applied WGCNA to all DEGs found in the combined datasets that were discovered from the differential expression analysis between the OA and control groups (|logFC| > 0 and *p* < 0.05). The hierarchical clustering tree was used to cluster the OA and control groups, with the grouping data annotated without a cut height specified. A screening criterion of 0.9 was then used to find the best number of modules. The DEGs between the investigated groups were then organized into distinct modules based on their co-expression patterns (**Figure 4A**). Afterwards, the DEGs were reclustered, and the connections between the genes and their respective new modules were shown (**Figure 4B**). After that, the cut height for merging modules was set to 0, and any modules having a cut height less than zero were combined (**Figure 4C**). According to the results, a correlation between OA and the seven modules was found to be significant (*p* < 0.05, |correlation| ≥ 0.3). The DEGs within these seven modules were then analyzed. By intersecting the previously identified 169 AAMRDEGs with the DEGs present in the seven modules, a Venn diagram was generated (**Figures 4D–J**) to identify module-specific AAMRDEGs. Seventy-eight DEGs linked to modular AAM were detected (**Supplementary Table S5**).

### 3.6 OA diagnosis model construction

**Figure 5:**
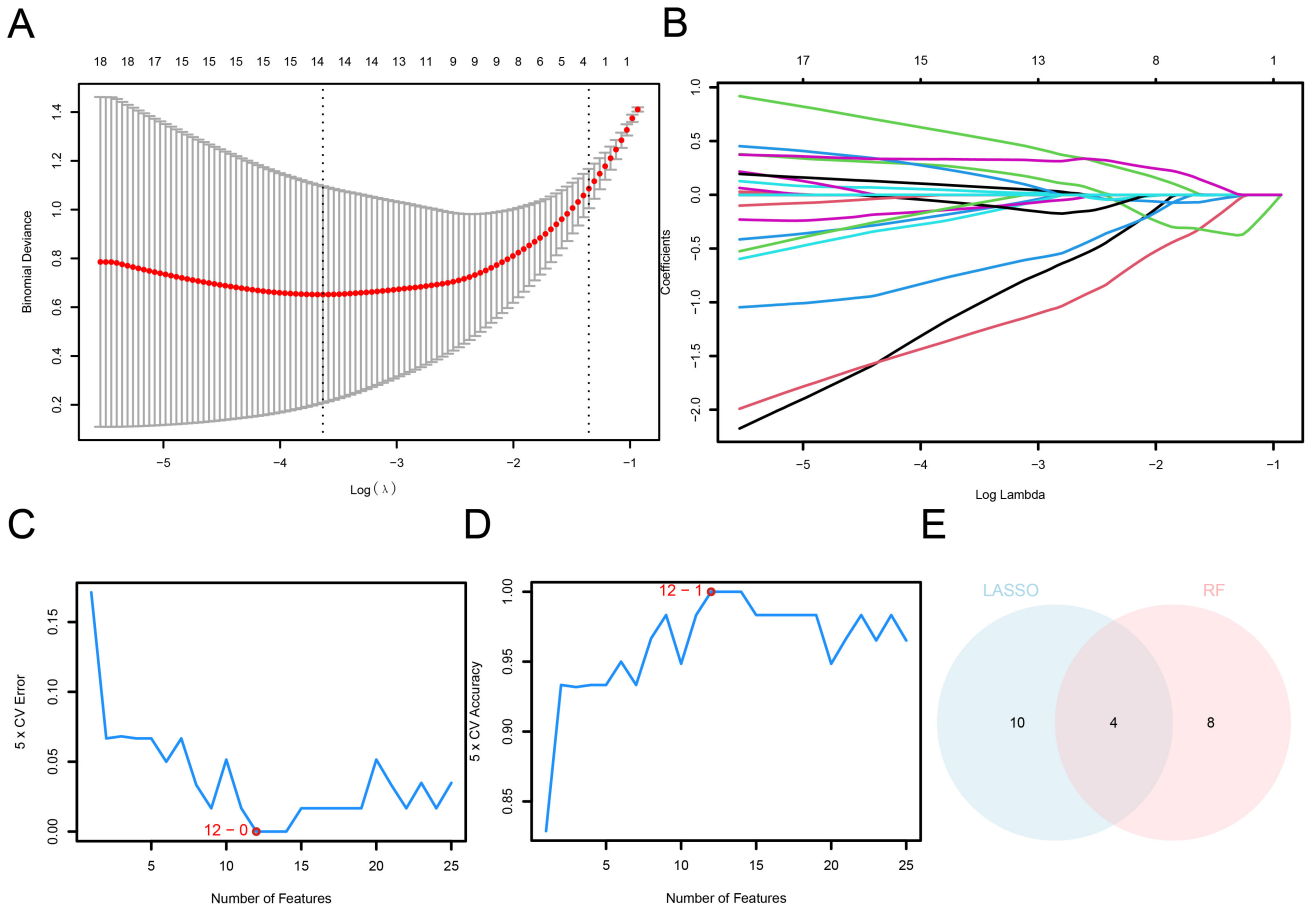
Construction of OA diagnostic model. **(A)** AAMRDEGs in combined datasets using the LASSO regression diagnosis model. **(B)** LASSO diagnosis model: Variable trajectory. **(C)** SVM algorithm: The count of genes with the minimum error rate. **(D)** SVM algorithm: The count of genes with the maximum accuracy. **(E)** Venn diagram: Intersection between LASSO and SVM algorithms.

Leveraging machine learning approaches, we developed LASSO and SVM-based diagnostic models using the selected AAMRDEGs. To estimate the diagnostic potential of 78 AAMRDEGs in OA, a univariate logistic regression model was developed using these genes. The analysis revealed that all 78 genes demonstrated statistical significance within the model (*p* < 0.05; **Supplementary Table S6**). Subsequently, a diagnostic model for OA was constructed via LASSO regression analysis based on the 78 AAMRDEGs. The results were visualized using the LASSO regression model diagram (**Figure 5A**) and the variable trajectory diagram (**Figure 5B**). The findings indicate that the model comprised 14 genes: *MTHFD2, PPP1R15A, GABBR1, GAMT, DPYS, SLC2A4, RPL3L, SLC6A11, CSN1S1, FBP1, RPL27A, IBA57, DNAJC12, and WNT5B.*

Then, an SVM model was built according to the 14 AAMRDEGs and the SVM algorithm. When the count of genes was optimized, the model achieved the minimum error rate (**Figure 5C**) and maximum accuracy (**Figure 5D**). The findings indicated that the highest accuracy of the SVM model was obtained when the count of genes was reduced to 12. The 12 identified AAMRDEGs include: *MTHFD2, PPP1R15A, WNT5B, BMP2, VEGFA, SLCO4A1, SLC7A5, SLC2A4, ETFB, SFTPD, ATF3, and MYC*.

AAMRDEGs derived from LASSO regression and SVM models were employed for the identification of key genes. By determining the intersection of these gene sets, four AAMRDEGs were identified as key candidates for further investigation. A Venn diagram illustrating this intersection is presented in **Figure 5E**. The four key genes that were identified were *MTHFD2, PPP1R15A, SLC2A4, and WNT5B*.

### 3.7 OA diagnosis model validation

**Figure 6:**
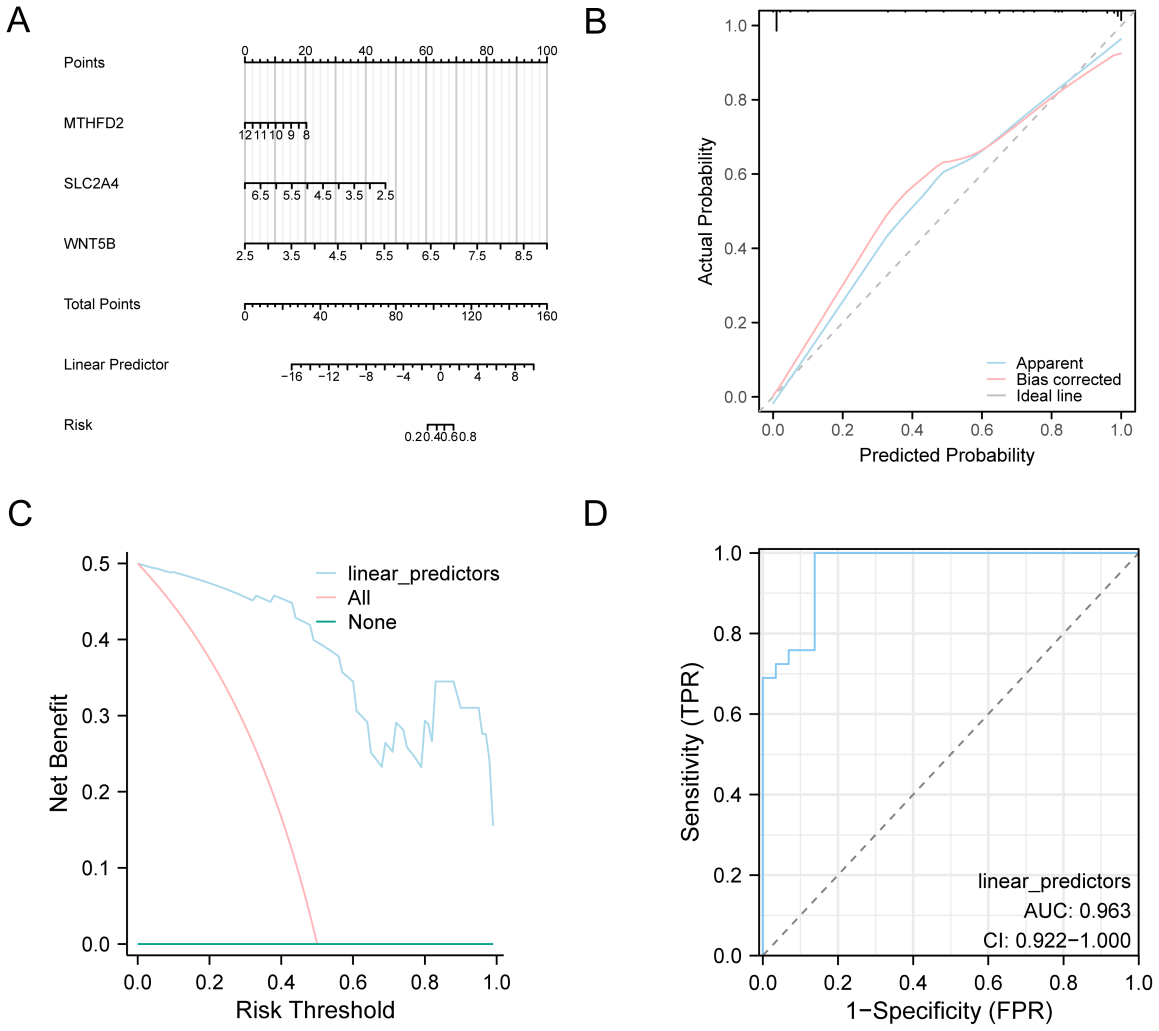
Diagnostic and validation analysis of OA. **(A)** Nomograms in combined datasets of key genes in OA diagnostic models. **(B)** OA diagnosis model based on combined datasets of key genes from the calibration curve, and **(C)** DCA. **(D)** Logistic regression model of linear predictors in the combined datasets of ROC analysis. The DCA y coordinate represents the net income figure, and the abscissa represents the probability threshold or threshold probability.

To systematically assess the reliability and clinical utility of the OA diagnostic model, we conducted a series of evaluation approaches including nomogram visualization, calibration analysis, DCA, and ROC curve assessment. A nomogram was constructed depending on the key genes to elucidate their relationships within the combined GEO dataset to improve the validation of the OA diagnostic model (**Figure 6A**). The results established that the model gene *WNT5B* expression level showed a significant elevation compared to other variables, underscoring its utility in the OA diagnostic model. Then, calibration analysis was employed to generate a calibration curve that was utilized to compare the model’s prediction performance with the actual outcomes by comparing the observed probabilities to the model’s predicted probabilities under numerous circumstances (**Figure 6B**). The results demonstrated that the calibration line deviated slightly from the ideal model’s diagonal but remained closely aligned with it. **Figure 6C** displays the results of our decision curve analysis, which we conducted using the model genes from the combined datasets to evaluate the OA diagnostic model’s clinical value. According to the results, the model’s curve always outperforms the “All Positive” and “All Negative” standards within a certain range, showing that it is more effective and provides a better net benefit. Additionally, we generated the ROC Curve for the logistic regression model’s linear predictors across various groups within the combined datasets, with the outcomes depicted in **Figure 6D**. This figure demonstrates that the logistic regression model exhibits robust diagnostic performance within the combined datasets.

### 3.8 PPI network construction

As interactions among diagnostic genes may reveal underlying molecular crosstalk in OA, we constructed PPI networks to identify potential functional associations. The PPIs of four key genes (*MTHFD2, PPP1R15A, SLC2A4*, and *WNT5B*) were assessed via the STRING database. The criteria implemented included the lowest required interaction score of > 0.150, which indicates low confidence, and a maximum of five interactors. Based on these criteria, a PPI network was constructed and visualized (**Supplementary Figure S4A**). Following this, we utilized the GeneMANIA website to identify potential genes that were related to the four key genes. Then, we constructed an interaction network to study the physical interactions, shared protein domains, and gene interactions (**Supplementary Figure S4B**).

### 3.9 RNA regulatory network construction

To gain insight into the upstream regulatory landscape of the diagnostic genes, we explored miRNA–mRNA and transcription factor–mRNA interactions using publicly available databases and network visualization. Here, we used the StarBase database to obtain 70 miRNAs linked to the four key genes (*MTHFD2*, *PPP1R15A*, *SLC2A4*, and *WNT5B*). Then, we built an mRNA-miRNA regulatory network and displayed it using Cytoscape (**Supplementary Figure S5A**). **Supplementary Table S7** provides detailed data. The ChIPBase database was also accessed to find TFs that were linked to these four key genes. Next, Cytoscape was deployed to build and display an mRNA-TF regulatory network. This network included 82 TFs and three key genes (*MTHFD2*, *PPP1R15A*, and *WNT5B*) (**Supplementary Figure S5B**). **Supplementary Table S8** lists detailed data.

### 3.10 Differential expression analysis of key genes between different groups

**Figure 7:**
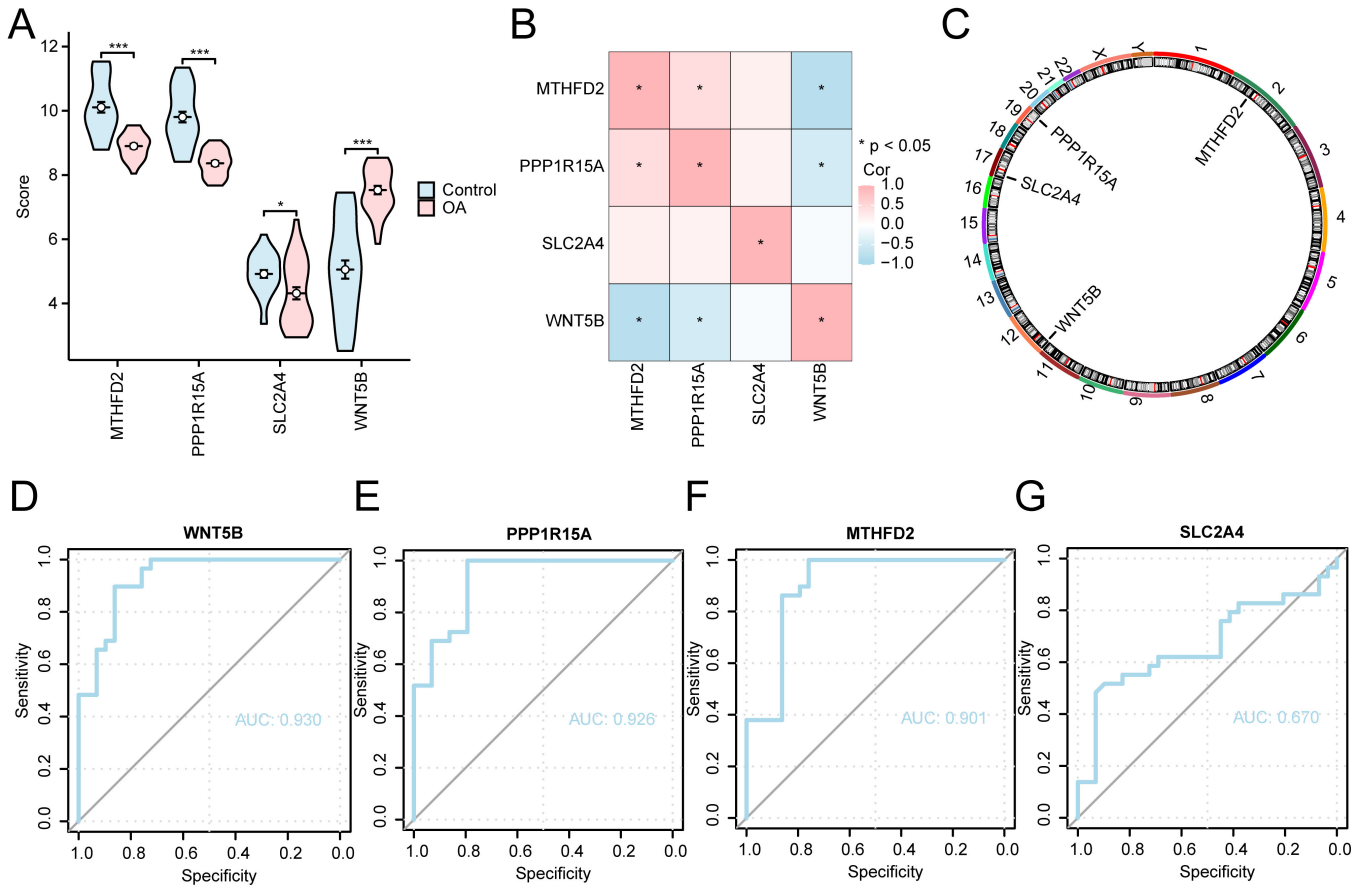
Key gene analysis of expression differences between different groups. **(A)** Group comparison plots of control and OA groups for the combined dataset. **(B)** Correlation analysis of key genes. **(C)** Chromosomal mapping of key human genes Key genes **(D)** WNT5B, **(E)** PPP1R15A, **(F)** MTHFD2, and **(G)** SLC2A4 in combined datasets of ROC curve analysis. *p < 0.001, *p < 0.05.

Expression differences of the key diagnostic genes were further examined across OA and control samples, accompanied by correlation analysis and ROC curve evaluation to assess their clinical predictive value. Our results revealed that three genes (*MTHFD2, PPP1R15A*, and *WNT5B*) exhibited significant differences in expression (*p* < 0.001), whereas *SLC2A4* demonstrated a moderately significant difference (*p* < 0.05, **Figure 7A**). A correlation analysis was conducted using the expression matrix of four key genes from the combined datasets, and a heatmap was employed to show the results (**Figure 7B**). This analysis illustrated a positive correlation between *PPP1R15A* and *MTHFD2*, whereas *WNT5B* was negatively correlated with both *MTHFD2* and *PPP1R15A*. Additionally, the chromosomal locations of the four genes were annotated and visualized (**Figure 7C**). *MTHFD2* is located on chromosome 2, *WNT5B* on chromosome 11, *SLC2A4* on chromosome 17, and *PPP1R15A* on chromosome 19. The ROC curves for the four key genes in the combined datasets were generated (**Figures 7D–G**). The expression differences of the key genes *MTHFD2*, *PPP1R15A*, and *WNT5B* demonstrated high discriminatory accuracy between several groups, with AUC > 0.9. Conversely, the difference in expression of *SLC2A4* exhibited lower discriminatory accuracy, with AUC values ranging between 0.5 and 0.7.

### 3.11 Immune infiltration analysis

**Figure 8:**
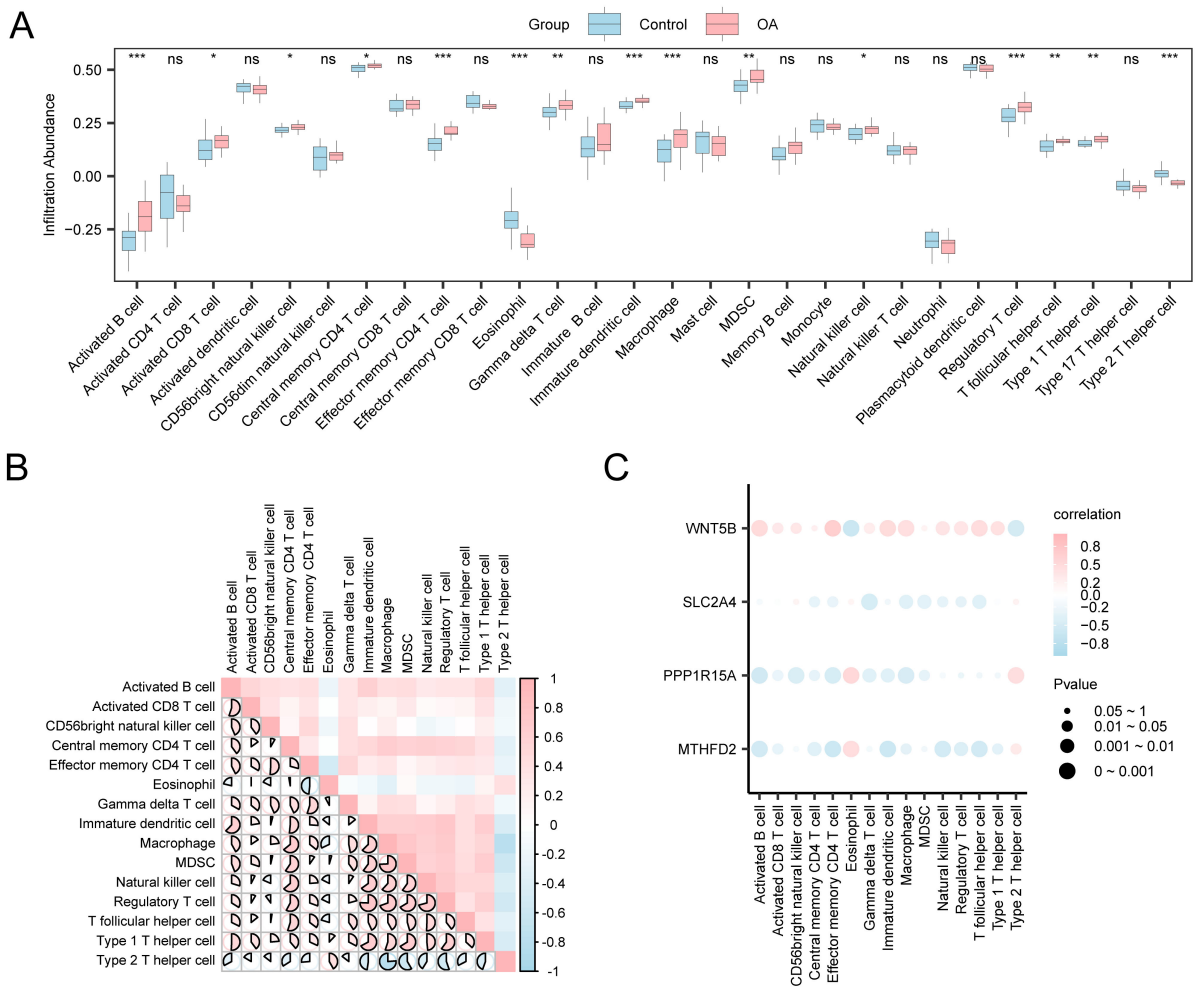
Immune infiltration analysis by ssGSEA algorithm. **(A)** Comparison chart of immune cells between control and OA groups. **(B)** Heatmap: Correlation of ICI abundance in combined datasets. **(C)** Bubble chart: Correlation between key genes and abundance of ICI in integrated datasets. ns: *p* ≥ 0.05, not statistically significant; *p < 0.05, **p < 0.01, ***p < 0.001.

Given the pivotal role of immune dysregulation in OA, this section evaluates immune cell infiltration patterns using ssGSEA and explores their associations with key gene expression. Using the expression matrix derived from the combined datasets, the ICI abundance of 28 distinct immune cell types was determined using the ssGSEA algorithm. First, a group comparison chart was generated to demonstrate the differential expression of ICI abundance across various groups. The group comparison chart (**Figure 8A**) revealed that 15 immune cell types exhibited statistically significant differences (*p* < 0.05), including activated B cells, activated CD8^+^T cells, and CD56^bright^ natural killer cells, among others. Subsequently, the correlation outcomes of the infiltration abundance of the 15 immune cell types in the combined datasets (**Figure 8B**). The outcomes show that there was a strong positive association among immune cells but a strong negative association among type 2 T helper cells and macrophages (r = −0.762, *p* < 0.05). Moreover, a correlation bubble chart illustrates the association between key genes and the abundance of ICI (**Figure 8C**). According to the correlation bubble chart, the majority of immune cells showed a strong relationship, with the gene *WNT5B* showing the most positive association with effector memory CD4 T cells (r = 0.662, *p* < 0.05).

### 3.12 OA subtypes construction

**Figure 9:**
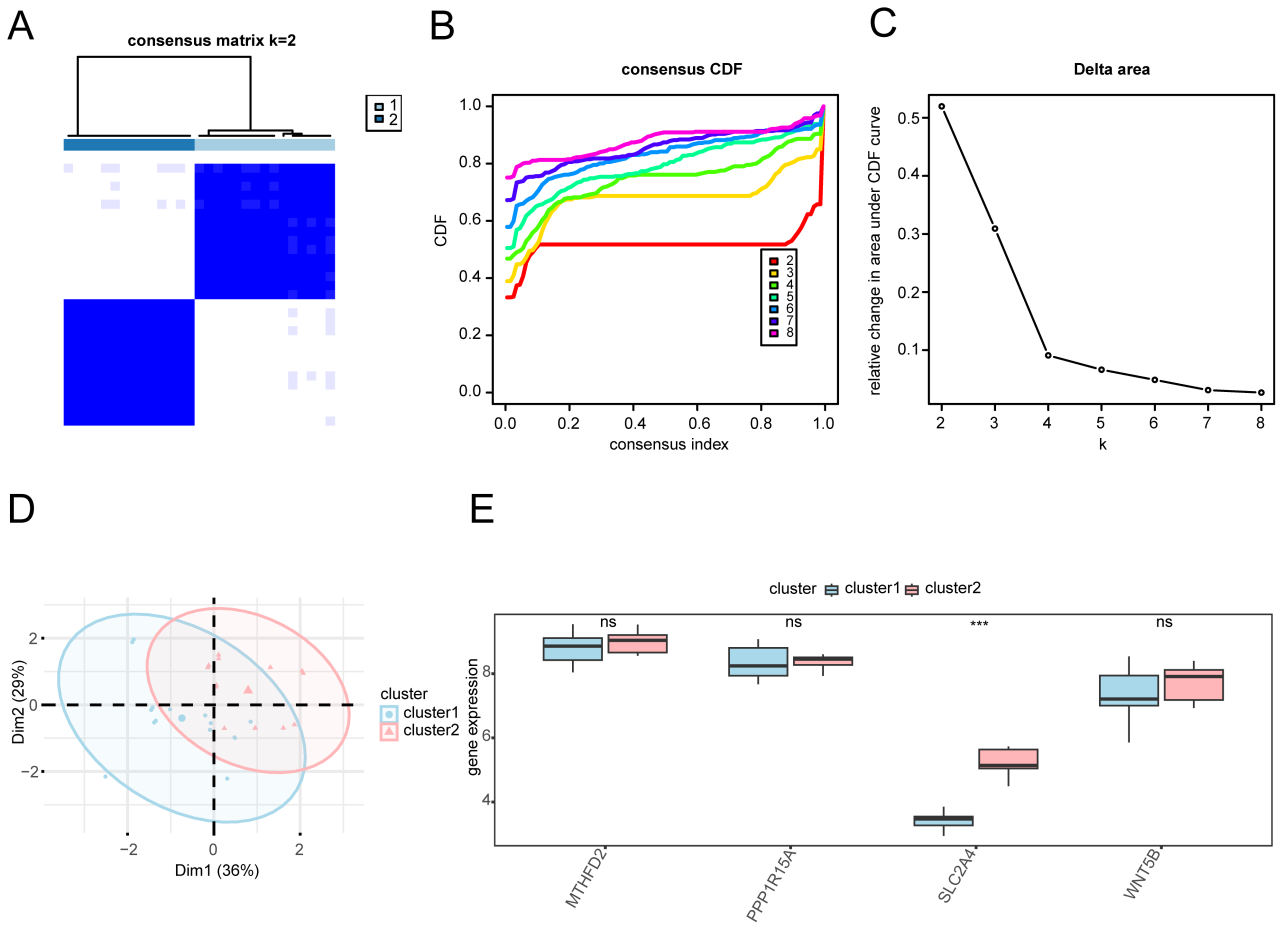
Consensus clustering analysis for OA. **(A)** Heatmap: Consistency clustering results matrix for OA samples. **(B)** Consistency cumulative distribution function and **(C)** Delta plot of consistency clustering analysis. **(D)** PCA plot: Two subtypes of OA. **(E)** Comparison chart of key genes in OA between the two subtypes. Subtype A (Cluster1) is depicted in blue, and subtype B (Cluster2) in pink. ***p < 0.001.

Based on the expression patterns of key genes, molecular subtypes of OA were identified through consensus clustering, followed by PCA and differential analysis to uncover their distinct biological characteristics. To determine the subtypes of OA within the sample cohort, the R package ConsensusClusterPlus was used to perform a consensus clustering analysis based on the levels of four key genes: *MTHFD2*, *PPP1R15A*, *SLC2A4*, and *WNT5B* (**Figures 9A–C**). This analysis delineated two distinct subtypes: A (cluster 1, comprising 15 samples) and B (cluster 2, comprising 14 samples). The PCA revealed significant differences between these subtypes (**Figure 9D**). Furthermore, a comparative analysis of the groups revealed a significant difference in the expression of the key gene *SLC2A4* between the subtypes (*p* < 0.001; **Figure 9E**).

### 3.13 IIA based on OA subtypes

**Figure 10:**
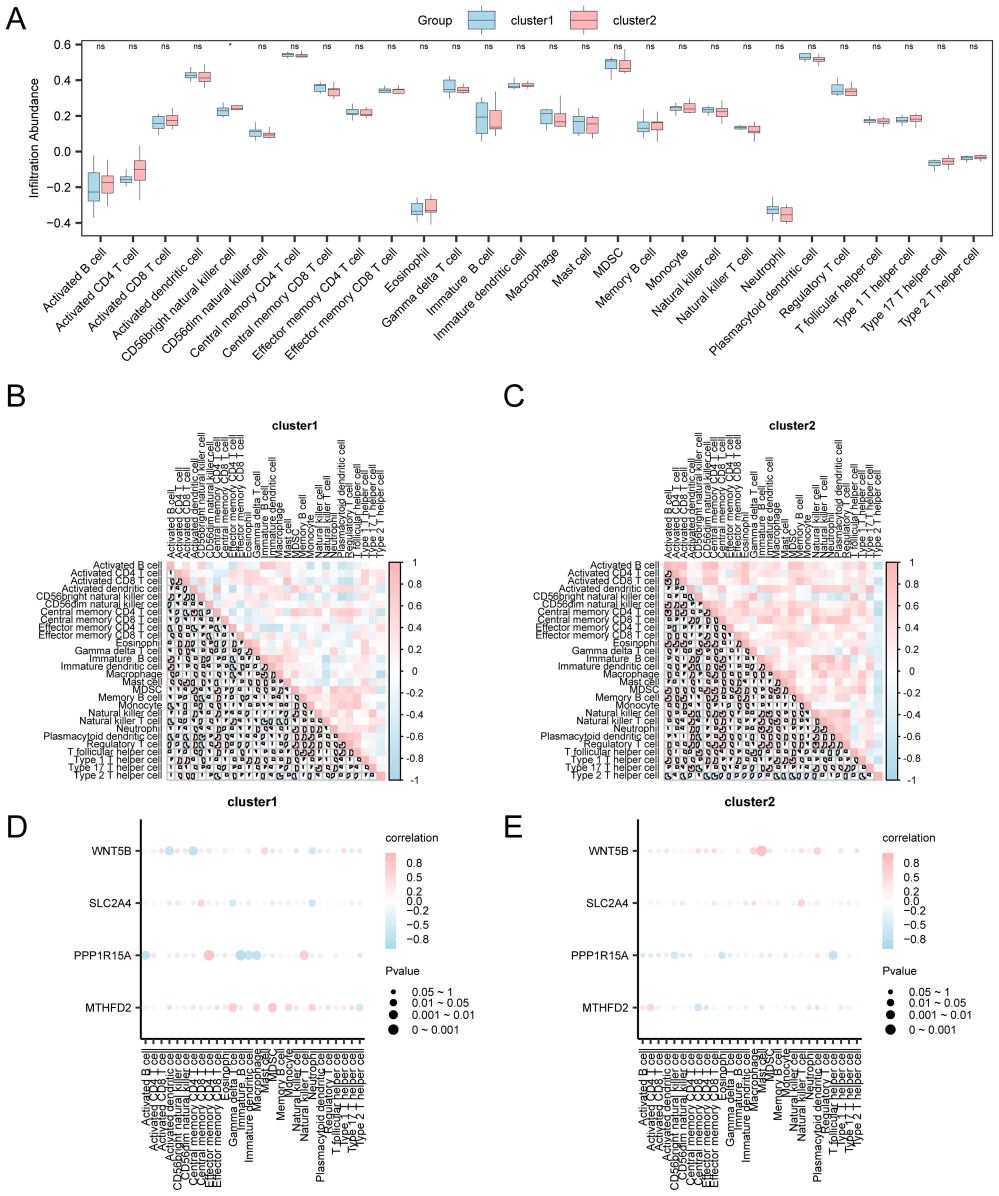
Risk group immune infiltration analysis by ssGSEA algorithm. **(A)** Comparison of immune cell grouping in Cluster1 and Cluster2 of OA samples. Results of correlation analysis of immune cell infiltration abundance in **(B)** Cluster 1 and **(C)** Cluster 2. Bubble plot of correlation between immune cell infiltration abundance and key genes in **(D)** Cluster1 and **(E)** Cluster2. ssGSEA: Single-Sample Gene-Set Enrichment Analysis; ns: *p* ≥ 0.05, not statistically significant; *p < 0.05, statistically significant. The absolute value of the correlation coefficient below 0.3 indicated weak or no correlation, 0.3–0.5 indicated weak correlation, 0.5–0.8 indicated moderate correlation, and > 0.8 indicated strong correlation. Blue represents Cluster 1, and pink represents Cluster 2. Pink represents a positive correlation, blue represents a negative correlation, and the depth of the color represents the strength of the correlation.

**Figure 11:**
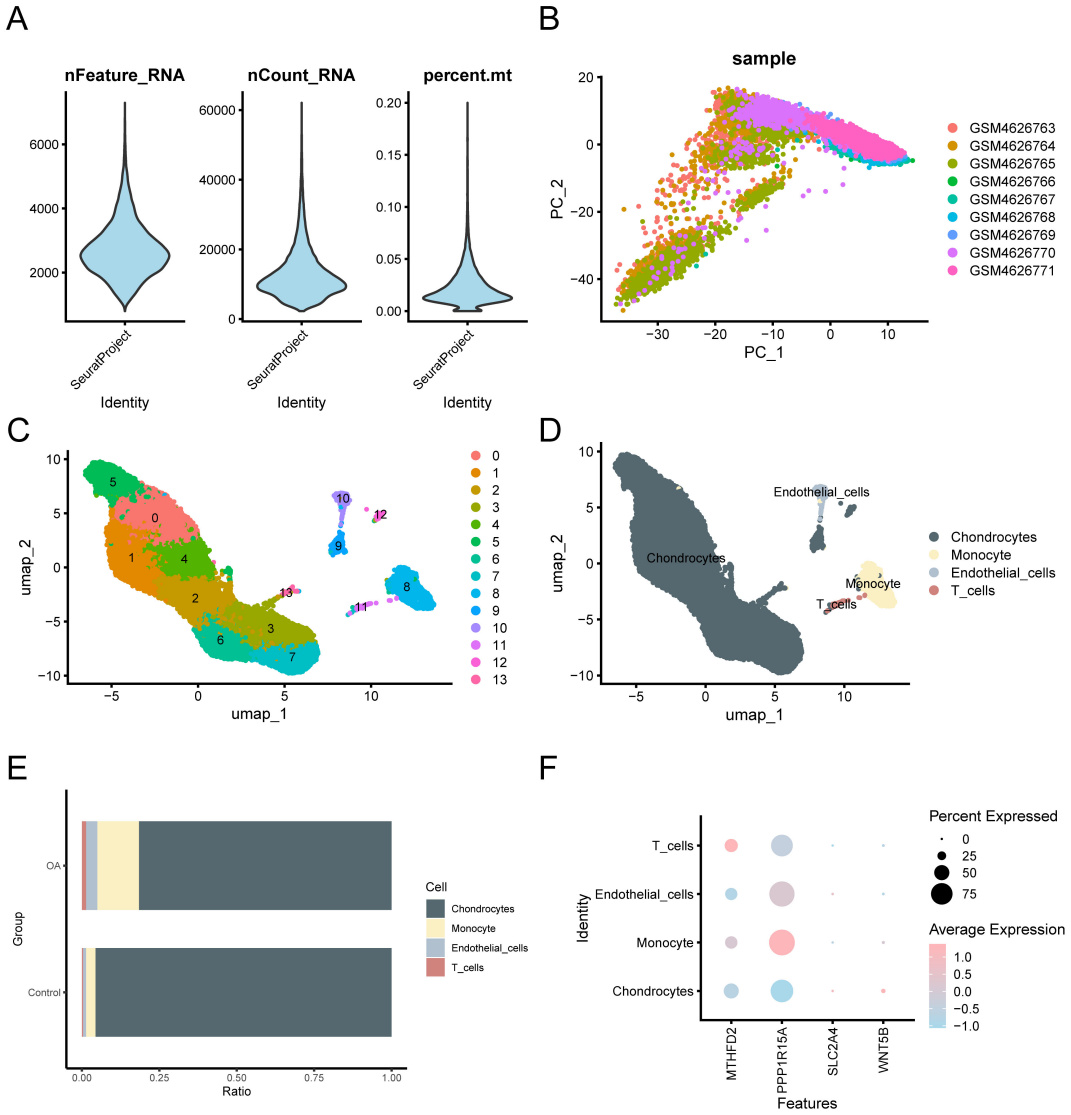
Quality control of GSE152805. **(A)** Violin plot of gene expression for the GSE152805 dataset. **(B)** Visualization of PCA of cell expression in different samples. **(C)** The cells were divided into 14 clusters using UMAP. **(D)** The cells were annotated into four cell types using cell marker genes: Chondrocytes, monocytes, endothelial cells, and T cells. **(E)** Bar graph of cell ratio between OA and control groups. **(F)** Bubble plot visualization of expression levels of key genes in the four cell types. The darker the color, the higher the expression level, and the larger the circle, the higher the expression proportion of genes within the cell population.

Building on the identified OA subtypes, we further investigated immune infiltration differences and their associations with key genes, aiming to delineate subtype-specific immune regulatory patterns. The expression matrix obtained from the combined datasets was employed to measure the abundance of 28 different immune cell types in OA samples via the ssGSEA technique. In order to illustrate how different groups’ expressions of ICI abundance varied, a group comparison chart was first constructed. One type of immune cell, CD56^bright^ natural killer cells, presented a significant difference (*p* < 0.05) according to the group comparison chart (**Figure 10A**). Then, a correlation heatmap was used to show the findings of the association between the infiltration abundance of 28 immune cell types in OA samples (**Figures 10B, C**). Cluster 1 of OA samples showed strong associations for the majority of immune cells. With an r-value of 0.879 and a *p* < 0.05, the most significant positive association was observed between activated dendritic cells and central memory CD4 T cells. In cluster 2, there was a highly significant positive association (r = 0.938, *p* < 0.05; **Figure 11C**) between the majority of immune cells and Tregs and natural killer cells. Furthermore, a correlation bubble chart (**Figures 10D, E**) shows the link between the infiltration abundance of four key genes and immune cells, showing that cluster 1 had the majority of immune cells with substantial correlations. Notably, *PPP1R15A* showed a significantly negative relationship with immature B cells (r = −0.829, *p* < 0.05). In contrast, the majority of immune cells in cluster 2 showed high associations, with the *WNT5B* gene showing a significant positive relationship with mast cells (r = 0.859, *p* < 0.05).

### 3.14 Quality control of single-cell dataset

**Figure 12:**
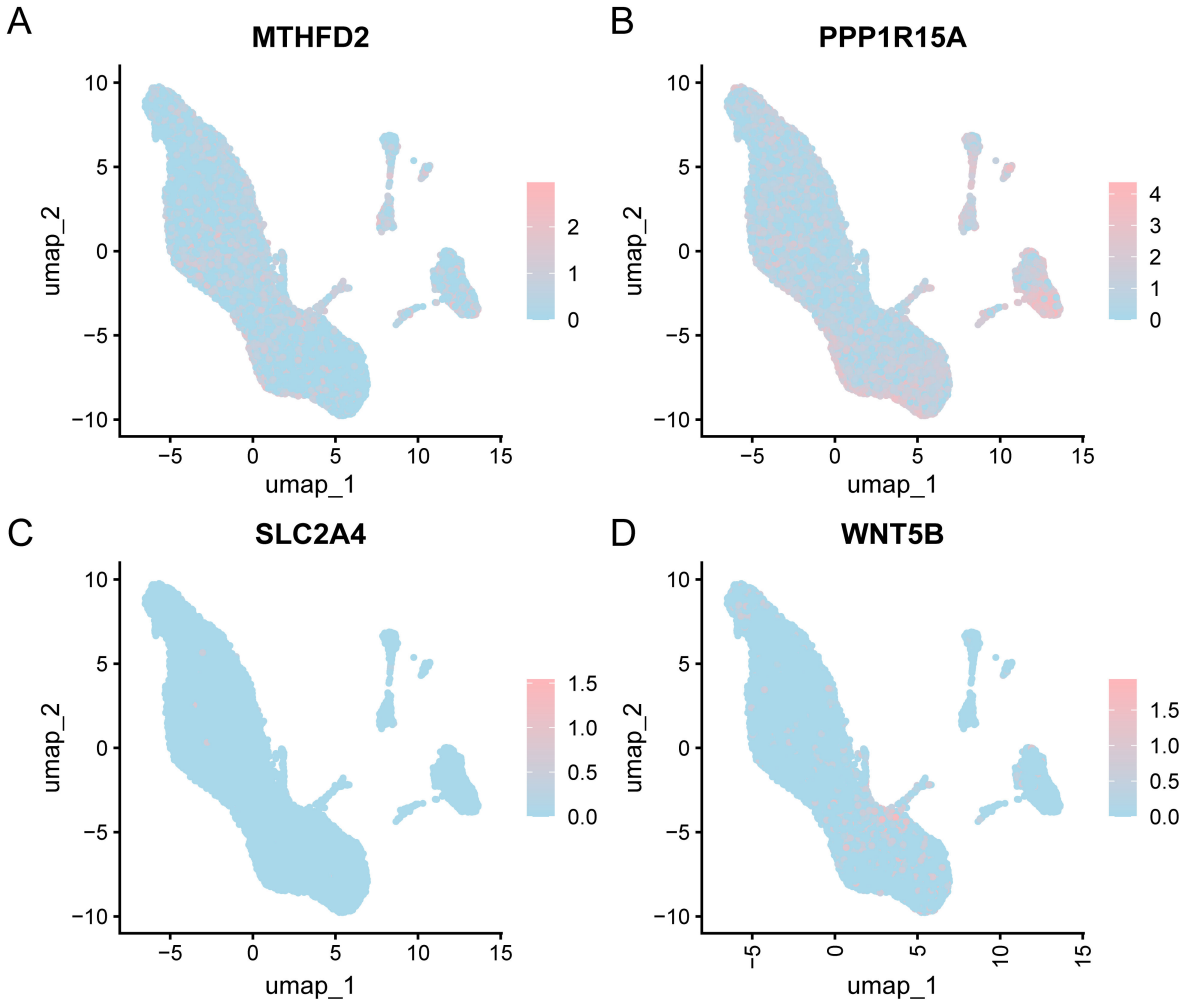
UMAP of key genes. UMAP depicting the expression levels of four key genes in the single-cell dataset: **(A)** MTHFD2, **(B)** PPP1R15A, **(C)** SLC2A4, and **(D)** WNT5B. The shade of pink represents the expression levels of genes in the single-cell dataset.

Prior to single-cell level analysis, we performed rigorous quality control, dimensionality reduction, and clustering of scRNA-seq data, followed by cell type annotation based on canonical marker genes. The “CreateSeuratObject” function from the Seurat v4.0 R package was used to import the counts matrix of three OA samples from the GSE152805 dataset. The import parameters included genes expressed in a minimum of three cells and cells expressing at least 200 genes. The distribution of gene features per cell and an average number of genes per UMI are illustrated in the violin plot (**Figure 11A**). Subsequently, quality control was carried out on the GSE152805 dataset, and cells with < 500 counts, log10GenesPerUMI < 0.8, or < 250 features were excluded. The cell expression profiles across different samples were analyzed using PCA (**Figure 11B**). Following the application of UMAP for dimensionality reduction with a resolution parameter set to 0.6, the cells were categorized into 14 clusters (**Figure 11C**). Four specific cell types were identified through manual annotation using cell marker genes (**Figure 11D**): Chondrocytes, monocytes, endothelial cells, and T cells. A bar chart illustrating the cell proportions across different groups (OA/control) (**Figure 11E**) indicated that the variations in cell proportions were minimal. The expression of the four key genes within the single-cell dataset is depicted using a bubble plot (**Figure 11F**) and UMAP plots (**Figures 12A–D**).

### 3.15 Differential genes of single-cell clusters and AUCell analysis

**Figure 13:**
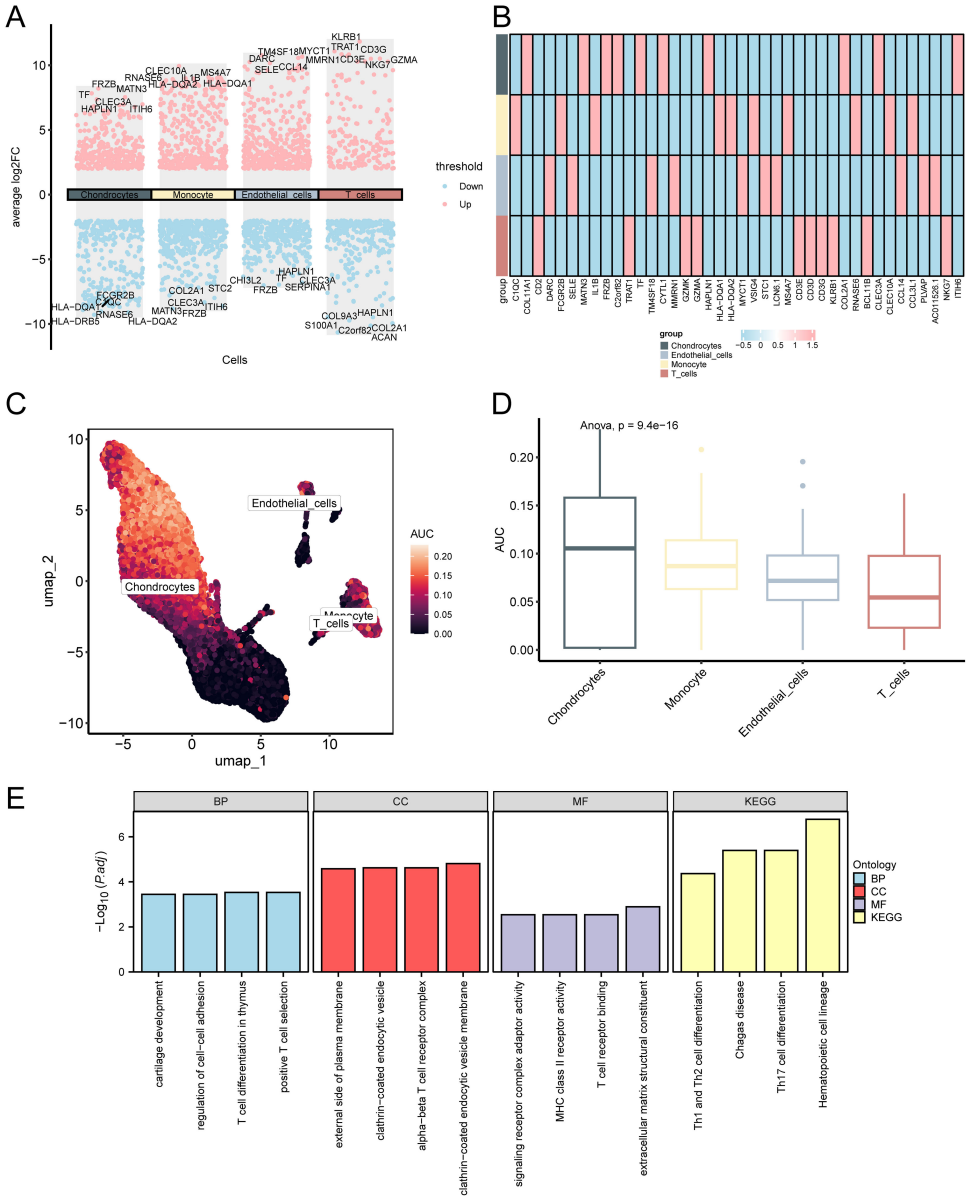
Differential gene expression and AUCell analysis for GSE152805. **(A)** . Volcano plot: Differential gene expression in cells. Pink denotes genes with elevated expression in that cell, while blue signifies genes with suppressed expression in that cell. **(B)**. Heatmap: Differential gene expression in a single cell group. The AUC score of the single-cell group differential genes was visualized using the **(C)** UMAP map and **(D)** group comparison map between different cell clusters. A lighter color on the UMAP map indicates a higher AUC score. **(E)** GO and KEGG enrichment of histogram analysis: Differential genes in single-celled groups: BP, CC, MF, and biological pathways. GO and KEGG terms are depicted on the abscissa.

Differentially expressed genes among cell types were identified, and AUCell scoring was applied to assess gene set activity across individual cells, offering insights into the distribution and potential functions of key genes at the single-cell level. Herein, we identified DEGs between cell types using the R package FindAllMarkers, applying thresholds of |logFC| > 2.00 and adjusted *p* < 0.05. A volcano plot was created to show these DEGs (**Figure 13A**). A heatmap was employed to depict the expression levels of the top 10 upregulated genes across cell types (**Figure 13B**). The outcomes illustrated that *FRZB, HLA−DRB5, and ITIH6* were predominantly expressed in chondrocytes; *FRZB, ITIH6, and COL2A1* were primarily expressed in monocytes; *FRZB, TM4SF, and SELE* were mainly expressed in endothelial cells; and *COL2A1, ACAN, and HAPLN1* were primarily expressed in T cells. The R package AUCell was employed to quantify the expression levels of the top 10 upregulated genes identified as single-cell differential genes for each cell within the GSE152805 dataset. The results are visualized using UMAP plots (**Figure 13C**) and group comparison plots (**Figure 13D**). Our findings indicated that chondrocytes exhibited the highest AUC score. To further explore the biological functions of these genes, we performed GO and KEGG enrichment analyses, and the results are shown in **Figure 13E**.

### 3.16 Verification of differential expression of four key genes using qRT-PCR analysis and IHC staining

**Figure 14:**
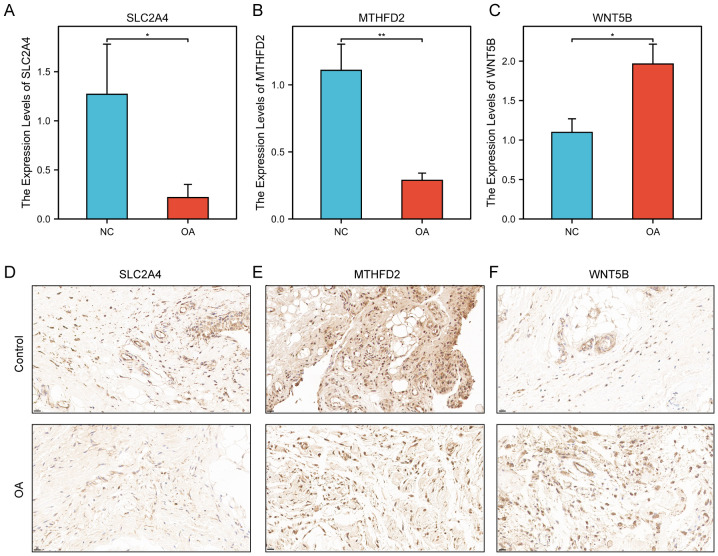
Experimental validation in clinical samples. **(A–C)** Relative mRNA expression levels of three key genes **(A)** *SLC2A4*, **(B)** *MTHFD2*, and **(C)** *WNT5B* in clinical samples. **(D–F)** Immunohistochemical staining: Expression of **(D)** SLC2A4, **(E)** MTHFD2, and **(F)** WNT5B in clinical tissue samples. The upper row corresponds to the control group, whereas the lower row pertains to the OA group. Positive staining is indicated by a brown-yellow coloration, signifying the localization of the target protein, while the nuclei are counterstained blue with hematoxylin. Bar = 20 μm. *p < 0.05, **p < 0.01.

Experimental validation was conducted using qRT-PCR and immunohistochemistry (IHC) on clinical synovial samples to confirm the differential expression and potential roles of the key genes in OA pathogenesis. Synovial tissues were harvested from the medial and lateral tibiofemoral compartments, as well as the suprapatellar regions. Samples from patients with osteoarthritis (n = 5) were collected during total knee arthroplasty, while control synovial samples (n = 5) were obtained from individuals undergoing arthroscopic procedures for non-inflammatory orthopedic conditions, devoid of clinical or histological evidence of active synovitis. Efforts were undertaken to ensure the comparability of anatomical sites across both groups. **Figures 14A–C** depict the outcomes of our qRT-PCR analysis. *MTHFD2, SLC2A4*, and *WNT5B* were identified as DEGs using bioinformatics and qRT-PCR analyses. The synovium of OA patients showed significantly lower levels of *MTHFD2* and *SLC2A4* expression compared to the control group (**Figures 14A, B** **),** but *WNT5B* expression was significantly higher (**Figure 14C**). Actin was applied as a reference gene to normalize the qRT-PCR expression levels of the candidate genes. **Figures 14D–F** demonstrate the outcomes of our IHC analysis. In alignment with the qRT-PCR results, relative to the control group, both MTHFD2 and SLC2A4 expression levels were significantly elevated in the OA synovium (**Figures 14D, E**), while the WNT5B expression level was significantly diminished (**Figure 14F**). In addition, the *PPP1R15A* expression level did not significantly differ between OA and control groups.

## 4 Discussion

On a global scale, OA is a prevalent joint disorder that considerably diminishes the quality of life of older people and imposes a considerable socioeconomic burden, as evidenced by the significant increase in healthcare expenditures due to increased medical consultations, therapeutic interventions, and rehabilitation services. In Spain, for instance, the healthcare burden associated with OA-related pain is significant, with expenses related to outpatient consultations constituting a major component (34). With its increasing incidence in the aging population, there is an imperative need for comprehensive research to improve diagnostic techniques and develop targeted therapeutic interventions.

Currently, therapeutic modalities for OA predominantly include pharmacological interventions, physical therapy, and surgical procedures (35, 36). However, these strategies only temporarily alleviate the symptoms and fail to fundamentally halt disease progression, with prolonged use potentially resulting in adverse effects (37). Consequently, it is imperative to thoroughly elucidate the pathological mechanisms of OA to devise more effective and targeted treatment strategies. The association between aberrant AAM and OA pathogenesis has attracted scholarly attention in recent years (38). Current research indicates that dysregulated AAM may significantly contribute to cartilage degradation and initiation of joint inflammation (39). However, the precise mechanisms underlying AAM during the pathological progression of OA remain poorly understood. Furthermore, the roles and regulatory networks of the associated DEGs remain to be elucidated, representing a significant gap in the existing body of research. This study aimed to identify AAMRDEGs and determine their roles in OA through comprehensive multi-level and multi-faceted bioinformatic analyses.

In this study, AAMRGs in OA were systematically analyzed. A subset of AAMRDEGs in OA was identified using differential expression analysis. The results of GO and KEGG analyses illustrated the predominant enrichment of these genes in different BP, including cellular AAM and catabolism. Our results demonstrated that amino acid synthesis is impaired in OA, thereby influencing various metabolic pathways associated with amino acids. Certain amino acids that are closely linked to the pathophysiology of OA, namely arginine, proline, and glutamic acid, are significantly affected. Arginine and proline are crucial for chondrocyte metabolism. Nitric oxide (NO), a metabolic product of arginine, contributes significantly to the inflammatory response in arthritis. The breakdown of the cartilage matrix and chondrocyte apoptosis may be caused by an excess of NO generation (40). Furthermore, proline metabolism is intricately related to the synthesis and breakdown of the cartilage matrix. Abnormalities in proline metabolism could compromise the structural and functional integrity of cartilage, thereby expediting the progression of OA. Additionally, existing literature indicates that glutamate influences the activity of osteoblasts and osteoclasts. Dysregulation of glutamate metabolism following the onset of OA might disrupt the dynamic equilibrium of bone remodeling, thereby exacerbating disease progression (41). Dysregulation of glutamate metabolism following the onset of OA might disrupt the dynamic equilibrium of bone remodeling, thereby exacerbating disease progression (39). Moreover, the pathways enriched in OA, such as cellular amino acid catabolic processes, provide insights into the biochemical alterations that might exacerbate cartilage degradation and inflammation. It is imperative to examine the interactions between AAMRDEGs and cellular pathways in cartilage and synovial tissues, as their dysregulation could initiate a cascade of events that contribute to OA progression. Elucidating these mechanisms could enhance our comprehension of OA pathology and potentially aid in the discovery of new treatment targets for modulating AAM.

Our diagnostic model, developed using LASSO regression and validated through SVM analysis, presents promising opportunities for early detection of OA. The identification of 14 AAMRDEGs as key diagnostic markers, including *MTHFD2*, *PPP1R15A*, and *WNT5B*, represents an innovative approach to OA diagnosis by harnessing molecular signatures to improve clinical decision-making. *MTHFD2* is a mitochondrial enzyme that has a critical function in folate and one-carbon metabolic pathways. Several malignancies have been linked to it, including glioblastoma, breast cancer, and hepatocellular carcinoma (42–44). However, its precise role in OA pathogenesis remains poorly understood. Yu et al. have identified *MTHFD2* as an apoptosis-associated gene that may influence OA progression[(44). Our results demonstrated that this influence is intricately associated with the disruption of AAM, particularly one-carbon metabolism. This disruption may have profound implications for the mitochondrial function of chondrocytes, potentially affecting cartilage proliferation and metabolic processes (45). Our research suggests that this influence is intricately associated with the disruption of AAM, particularly one-carbon metabolism. This disruption may have profound implications for the mitochondrial function of chondrocytes, potentially affecting both cartilage proliferation and metabolic processes (46). The *SLC2A4*, a key glucose transporter in muscle and adipose tissues, is essential for glucose metabolism and energy homeostasis. Initially, we observed a reduction in *SLC2A4* expression in patients with arthritis, which was potentially linked to the dysregulation of AAM signaling pathways. In OA patients, the reduced *SLC2A4* expression in the cartilage might impair glucose metabolism, adversely affecting chondrocyte function and viability. This deficiency was associated with increased chondrocyte apoptosis and inflammation. Furthermore, *SLC2A4* dysfunction might result in decreased synthesis of the cartilage matrix, thereby affecting the structural integrity and functionality of the joints (47). The *PPP1R15A* is a regulatory subunit of the intracellular eIF2α phosphatase, playing a critical role in modulating cellular stress responses. By facilitating the dephosphorylation of eIF2α, it aids in the resolution of the integrated stress response, thereby influencing apoptotic pathways (48). Although its specific role in OA remains unexplored, *PPP1R15A* levels decrease following the onset of OA. This reduction might contribute to the dysregulated cellular stress responses, potentially leading to chondrocyte apoptosis and subsequent joint tissue damage. *WNT5B*, a member of the WNT family, participates in the non-canonical WNT signaling pathway, which is independent of β-catenin and often antagonizes canonical WNT signaling. Several physiological activities, such as cell migration, proliferation, and differentiation, have been linked to this protein (49). Recent investigations have demonstrated that *WNT5B* might serve as a critical node in the inflammatory signaling pathways that exacerbate OA pathology. Specifically, miR-140-3p and circ-PREX1 modulate *WNT5B* expression, thereby influencing chondrocyte apoptosis and inflammatory responses (50). Furthermore, *WNT5B* has been considered as a target of miR-374a-3p, and its downregulation mitigates lipopolysaccharide-induced damage in chondrocytes, underscoring its involvement in inflammatory pathways (51). Our results demonstrate that *WNT5B* may be involved in the dysregulation of AAM, potentially intensifying the inflammatory response observed in arthritis. To improve predictive capabilities further, future research should prioritize the optimization of model parameters and the exploration of integrating additional biomarkers, particularly those identified through metabolomic profiling of OA. The translation of this diagnostic model into clinical practice could revolutionize OA management, shifting the healthcare paradigm towards a more personalized and proactive approach.

The PPI network analysis revealed significant interactions among the key AAMRDEGs, thereby laying the foundation for understanding the molecular interactions that underlie OA pathogenesis. The notable association between *MTHFD2*, *PPP1R15A*, and *SLC2A4* indicates the existence of a highly regulated network, with each gene potentially interacting with ATF4. The ATF4 is an essential TF in the endogenous stress response, primarily facilitating cellular adaptation to environmental stressors. It has a crucial function in chondrocyte survival by modulating the endoplasmic reticulum stress and apoptotic pathways (52, 53). These observations imply that these key genes might interact with ATF4 to orchestrate essential processes like metabolism and inflammation in arthritis. These interactions offer valuable visions into the techniques by which perturbations in these networks might facilitate OA progression.

Recent studies have demonstrated that dysregulated amino acid metabolism significantly impacts immune cell function and infiltration, thereby shaping the immunological microenvironment in OA. For instance, arginine metabolism is crucial for regulating T cell proliferation and cytokine production, and its depletion can impair T cell receptor signaling and suppress immune responses. Similarly, the availability of glutamine is essential for the differentiation and function of Th17 cells, which play a pivotal role in OA-associated inflammation (54). Additionally, tryptophan catabolism via the kynurenine pathway influences immune homeostasis by promoting the development of regulatory T cells and suppressing effector responses (55). These mechanisms indicate that alterations in amino acid metabolism may drive immune cell infiltration and polarization within OA joints. In line with these mechanistic insights, our study investigated the relationship between amino acid metabolism–related gene expression and immune cell infiltration in OA synovial tissues.

By analyzing gene–immune correlations, we sought to further understand how amino acid metabolic alterations might modulate the local immune microenvironment in OA. The immunological landscape of OA is a key aspect elucidated by our findings. T cells are critically involved in OA pathogenesis. Empirical evidence indicates that the T cell stimulation and proliferation are intimately related to the inflammatory response characteristic of OA, with pronounced polarization towards the proinflammatory Th1 subset (56). This phenomenon was corroborated by our IIA, which revealed a significant increase in effector memory T cells, central memory T cells, γδ T cells, Tregs, and Th1 cells in OA patients. These infiltrating T cells not only secrete numerous proinflammatory cytokines, such as tumor necrosis factor α and interleukin-17 but also augment the activity of other immune cells by promoting intercellular interactions, thereby intensifying joint inflammation and damage (57). The interaction between *WNT5B* and immune cells, particularly CD4 T cells, is of particular interest. CD4 T cells are pivotal in orchestrating immune responses, and their effector memory subset responds rapidly upon re-exposure to antigens. The positive relationship between *WNT5B* expression and effector memory CD4 T cells suggests that *WNT5B* might be involved in the stimulation and proliferation of these cells within the osteoarthritic milieu. This hypothesis is further supported by current investigations that have reported the polarization of proinflammatory T cells in the peripheral blood of individuals with early-stage knee OA, demonstrating that *WNT5B* may have a function in T cell polarization (56). Consequently, investigating the immune regulatory interactions between *WNT5B* and T cells could be a novel therapeutic approach for treating OA. The role of B cells is of comparable importance. Recent studies indicate that B cells have a dual function, involved in humoral immune responses and the regulation of cartilage repair mechanisms via the secretion of antibodies and cytokines. B cells can produce specific antibodies that neutralize proinflammatory factors, thereby mitigating the inflammatory response (58). Conversely, hyperactivation of B cells might induce autoimmune responses, exacerbating cartilage damage (59). The increase in macrophages during IIA in patients with OA might be attributed to the substantial recruitment of chemokines. Macrophages can be categorized into M1 and M2 phenotypes, where M1 macrophages mainly facilitate proinflammatory responses, and M2 macrophages participate in tissue repair and anti-inflammatory processes (60, 61). The polarization state of these macrophages directly influences the inflammatory response and cartilage degradation. An increased number of M1 macrophages, which are linked to faster cartilage degradation and worsening joint inflammation, is found in the joints of OA individuals. The potential of immune modulation as a therapeutic strategy could be elucidated by investigating the dynamics of immune cells across various stages of OA. Furthermore, identifying specific immunological profiles associated with varying severities of OA could inform targeted interventions aimed at restoring homeostasis within the immune microenvironment, potentially mitigating the inflammatory aspects of OA. Using immunosuppressants or biological agents to reduce the secretion of proinflammatory factors might be an effective approach for modulating the immune response in OA. However, it is imperative to comprehensively evaluate the long-term effects and safety of these interventions (62).

This research has enhanced our comprehension of the role of AAMRDEGs in OA. Despite the application of batch effect correction measures during data processing, the complete elimination of potential inter-batch differences requires further validation. Moreover, our diagnostic model was developed and validated using a combined GEO dataset and has not yet been evaluated in an independent external cohort. As a result, its generalizability and applicability in real-world clinical settings require further assessment. Future research should prioritize ensuring the stability and reproducibility of the results by employing more rigorous multi-batch data integration methods. It should be noted that, the principal conclusions of this study are predominantly based on transcriptomic analyses of synovial tissue and multi-omics bioinformatic correlation analyses. However, there is a significant absence of direct functional and mechanistic experimental validation. Furthermore, the expression profiles examined were restricted to synovial tissue, despite OA impacting the entire joint, including cartilage and subchondral bone. Future investigations should aim to validate these findings across various joint compartments and further elucidate their functional relevance through both *in vitro* and *in vivo* experiments. The specific molecular mechanisms and regulatory networks involving key genes such as MTHFD2, SLC2A4, and WNT5B in OA remain to be comprehensively elucidated. Subsequent research should utilize cellular and animal models to systematically investigate the functional roles of these genes in modulating chondrocyte metabolism, apoptosis, inflammatory signaling, and immune cell interactions. Additionally, investigating their downstream signaling pathways and interactions with the immune microenvironment may offer a more robust theoretical and experimental basis for precise molecular subtyping and targeted therapy of OA.

In conclusion, this study was initiated with metabolomics of clinical samples, identifying a series of significant molecular markers closely associated with OA through comprehensive multi-level data integration and analysis. Consequently, an efficient and accurate diagnostic model was developed. These results improve our understanding of the pathogenesis of OA and its intricate regulatory networks and offer a solid foundation for future precision medical.

## Data Availability

The datasets presented in this study can be found in online repositories. The names of the repository/repositories and accession number(s) can be found in the article/**Supplementary Material**.
