# Supplementary material for: Exploration of differential expression and biological significance of amino acid metabolism genes in osteoarthritis
Source: Front Immunol. 2025 Jul 14;16:1588072. doi: 10.3389/fimmu.2025.1588072 (PMC12301216; doi:10.3389/fimmu.2025.1588072)
Supplement: Supplementary file 1 [file DataSheet1.pdf]

frontiers

Supplementary Material

1 Supplementary Figures and Tables

1.1 Supplementary Figures

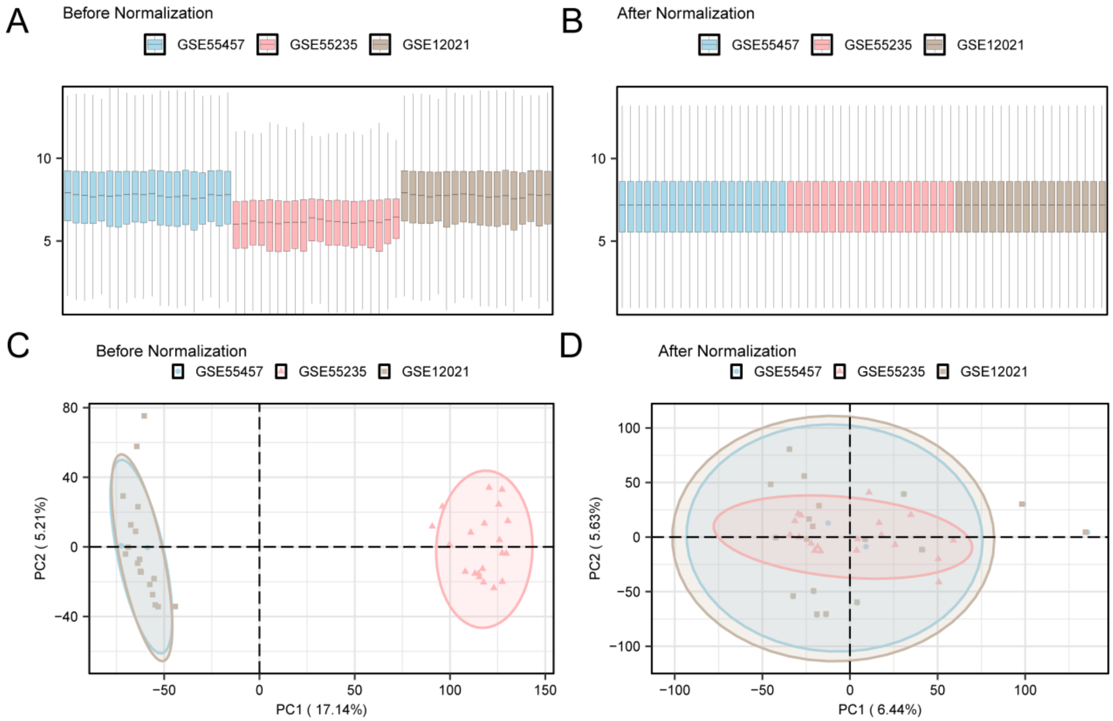

Supplementary Figure 1. Batch Effects Removal of GSE55457, GSE55235, and GSE12021. (A) Box plot of combined GEO datasets distribution before batch removal. **(B)** Post-batch combined datasets distribution boxplots. **(C)** PCA plot of the datasets before de-batching. **(D)** PCA map of the combined GEO datasets after batch processing.

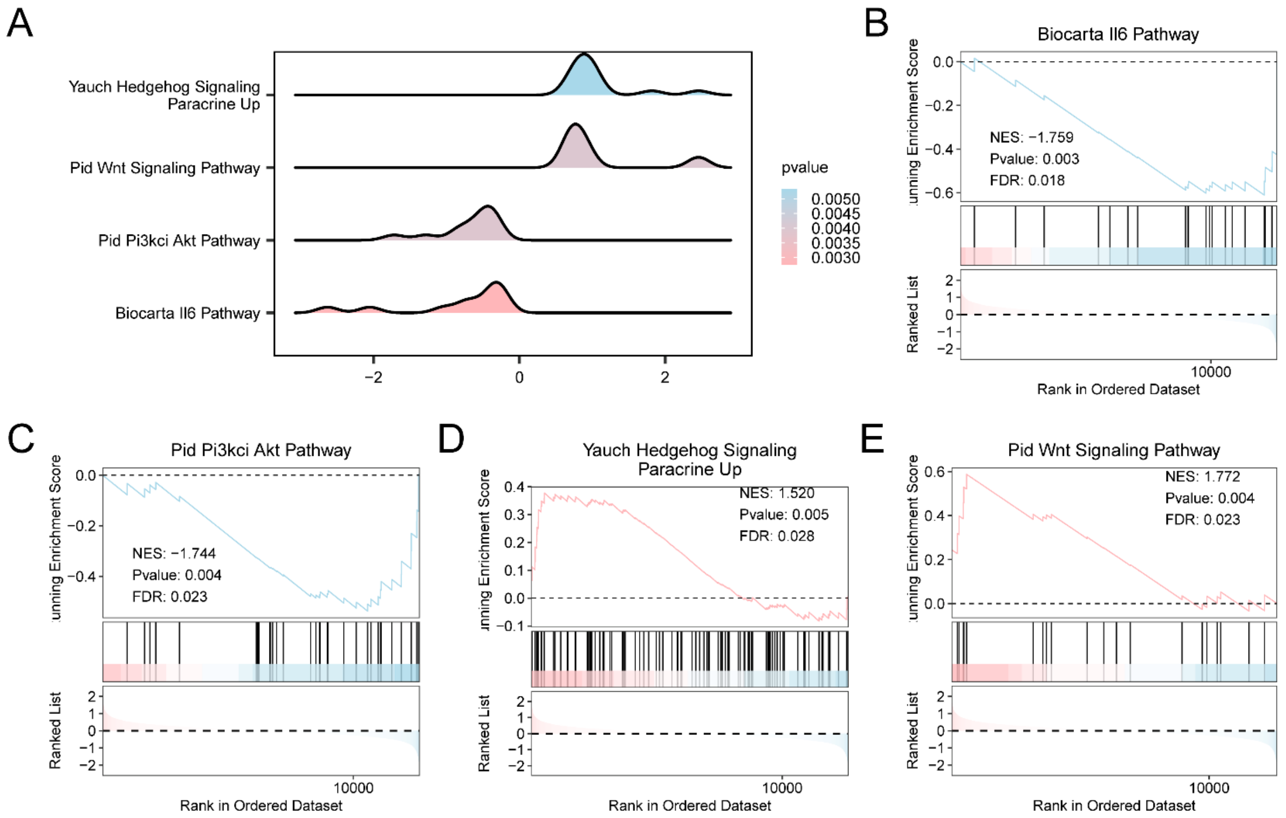

Supplementary Figure 2. Differential Gene Expression Analysis and GSEA for Combined Datasets. (A) GSEA biological functions mountain map of the combined GEO datasets. **(B-E)** GSEArevealed that combined datasets were significantly enriched in (B) IL6 Pathway, (C) Pi3kci Akt pathway, (D) Yauch Hedgehog Signaling Paracrine Up, and (E) Pid Wnt signaling pathway. In the mountain plot, the color represents the p-value; the redder p-value is smaller, and the bluer p-value is larger. The screening criteria of GSEA included $p < 0.05$ and FDR <0.05. Benjamini-Hochberg method was used for p-value correction.

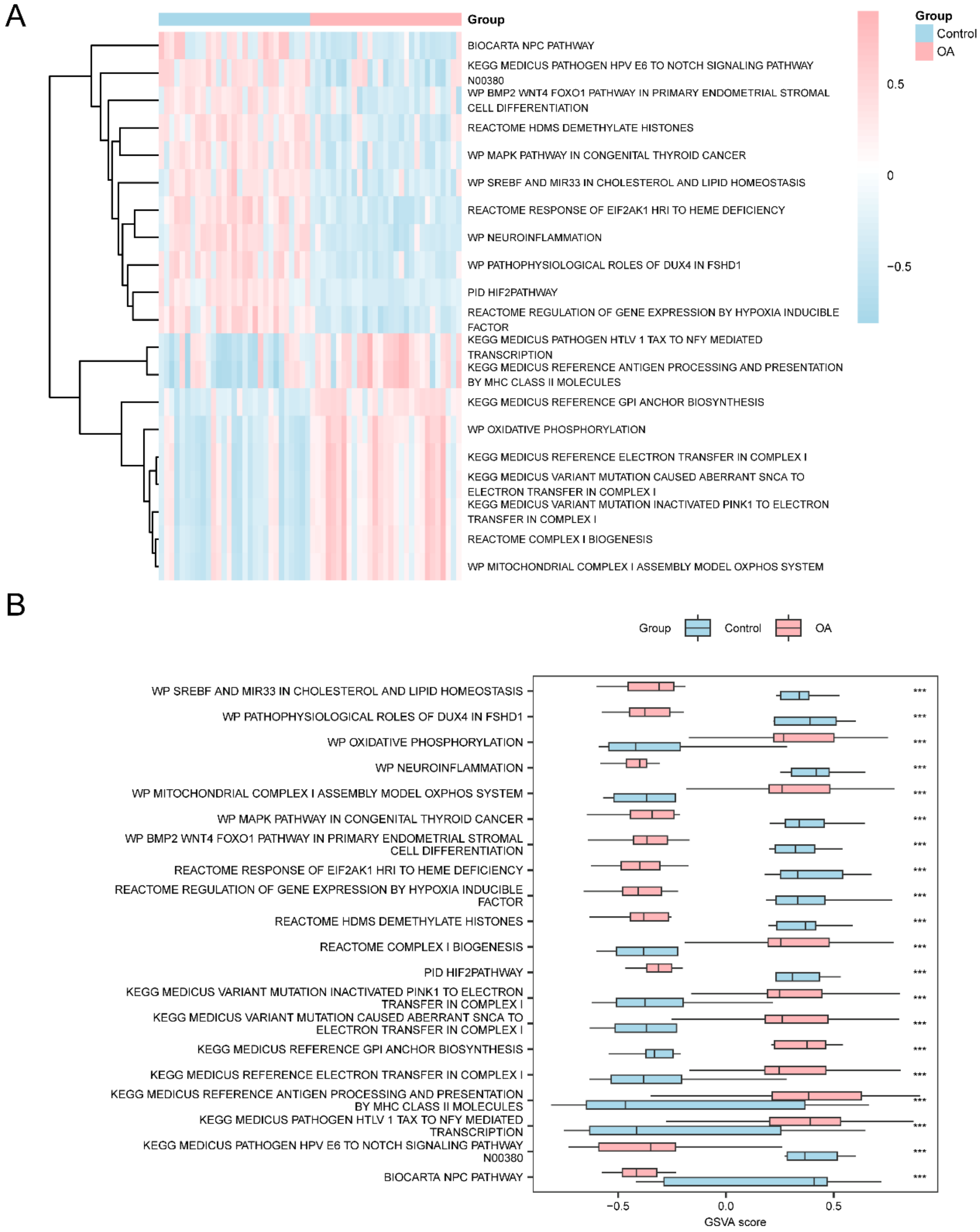

Supplementary Figure 3. Gene Set Variation Analysis. (A) Heatmap and **(B)** group comparison map of GSVA results between OA and control groups in the combined datasets. Pink represents the OA group, and blue represents the control group. The screening criteria for GSVA included $p < 0.05$, and the Benjamini-Hochberg method was used for *p-value* correction. In the heatmap, blue represents low enrichment, and pink represents high enrichment.

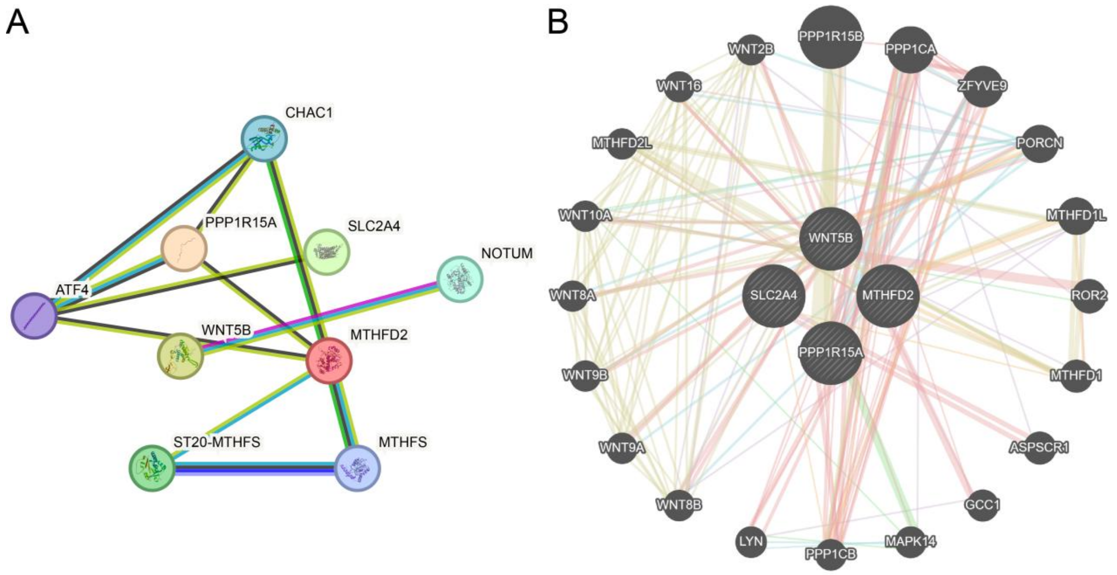

Supplementary Figure 4. Protein-protein Interaction Network. (A) PPI network of key genes. **(B)** Key genes predict the interaction network of genes with similar functions. Circular nodes represent genes, and the size is determined by the attributes and characteristics of the genes. Lines represent relationships, interactions, or functional connections between genes, and line thicknesses represent strong associations or important interactions.

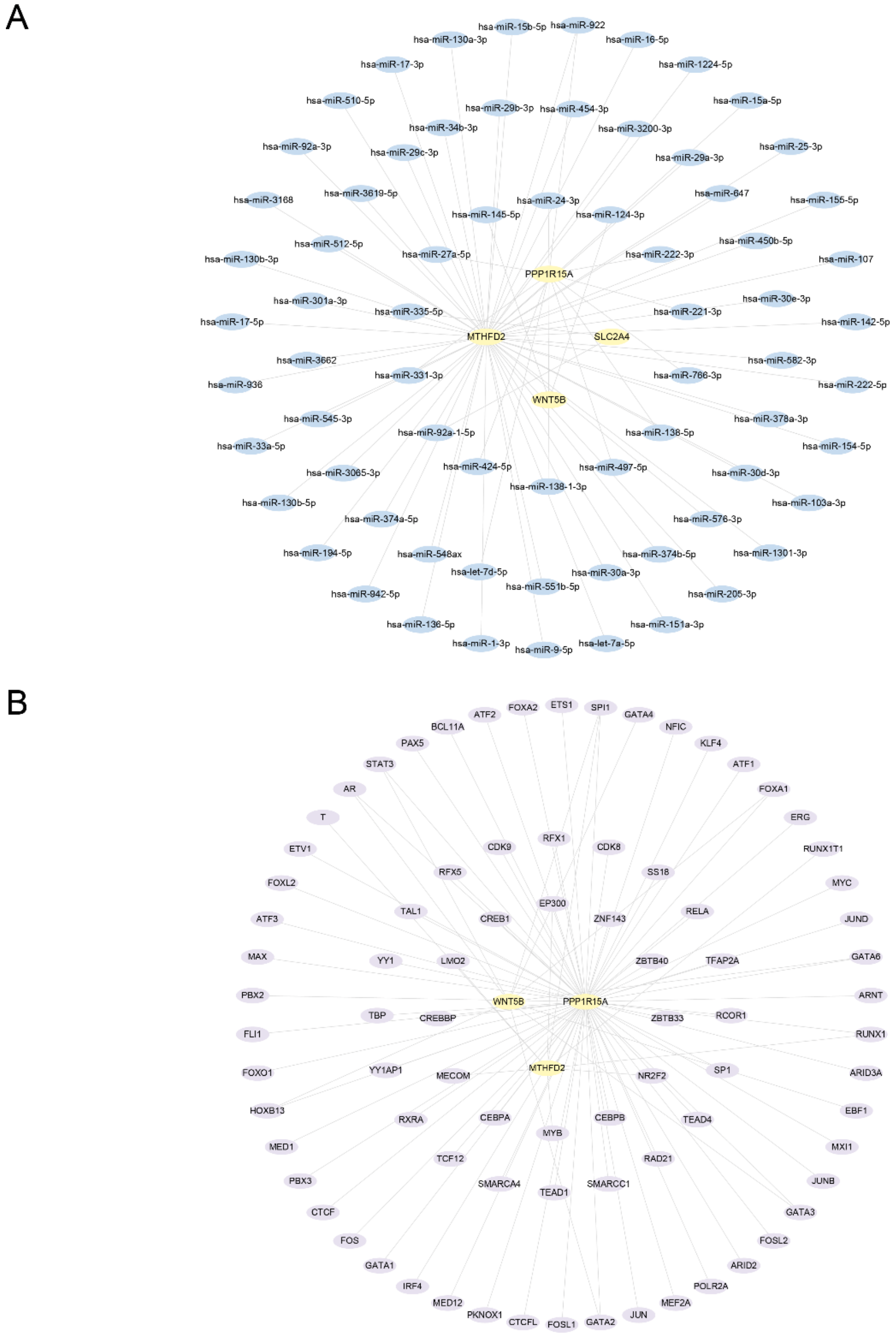

Supplementary Figure 5. Regulatory Network of Key Genes. (A) The mRNA-miRNA regulatory network of key genes. **(B)** mRNA-TF regulatory network of key genes.
