# Supplementary material for: Exploration of differential expression and biological significance of amino acid metabolism genes in osteoarthritis
Source: Front Immunol. 2025 Jul 14;16:1588072. doi: 10.3389/fimmu.2025.1588072 (PMC12301216; doi:10.3389/fimmu.2025.1588072)
Supplement: Supplementary file 2 [file Table1.docx]

Supplementary Material

# Supplementary Table 1

### Table S1 GEO Microarray Chip Information

|  | GSE55457 | GSE55235 | GSE12021 |
| --- | --- | --- | --- |
| Platform | GPL96 | GPL96 | GPL96 |
| Species | Homo sapiens | Homo sapiens | Homo sapiens |
| Tissue | Synovial Membrane Tissues | Synovial Membrane Tissue | Synovial Membrane Tissue |
| Samples in OA group | 10 | 10 | 9 |
| Samples in Control group | 10 | 10 | 9 |
| Reference | PMID:24690414 | PMID:24690414 | PMID:18721452 |

GEO，Gene Expression Omnibus；OA，Osteoarthritis.
