# Supplementary material for: Exploration of differential expression and biological significance of amino acid metabolism genes in osteoarthritis
Source: Front Immunol. 2025 Jul 14;16:1588072. doi: 10.3389/fimmu.2025.1588072 (PMC12301216; doi:10.3389/fimmu.2025.1588072)
Supplement: Supplementary file 4 [file Table3.docx]

Supplementary Material

# Supplementary Table 3

### Table S3. Results of GSEA for Combined Datasets.

| ID | setSize | enrichmentScore | NES | pvalue | p.adjust | qvalue |
| --- | --- | --- | --- | --- | --- | --- |
| BIOCARTA_IL6_PATHWAY | 21 | -0.610196299 | -1.758567121 | 0.002762139 | 0.023539109 | 0.018090726 |
| PID_PI3KCI_AKT_PATHWAY | 33 | -0.537785873 | -1.743673457 | 0.003931346 | 0.030135117 | 0.023160017 |
| YAUCH_HEDGEHOG_SIGNALING_PARACRINE_UP | 100 | 0.377483368 | 1.519587953 | 0.005331456 | 0.036912527 | 0.028368721 |
| PID_WNT_SIGNALING_PATHWAY | 22 | 0.587781303 | 1.772468181 | 0.003973293 | 0.030296357 | 0.023283935 |

GSEA：Gene Set Enrichment Analysis.
