# Supplementary material for: Exploration of differential expression and biological significance of amino acid metabolism genes in osteoarthritis
Source: Front Immunol. 2025 Jul 14;16:1588072. doi: 10.3389/fimmu.2025.1588072 (PMC12301216; doi:10.3389/fimmu.2025.1588072)
Supplement: Supplementary file 5 [file Table4.docx]

Supplementary Material

# Supplementary Table 4

### Table S4. Results of GSVA for Combined Datasets.

| Pathway | logFC | AveExpr | t | P.Value | adj.P.Val | B |
| --- | --- | --- | --- | --- | --- | --- |
| REACTOME RESPONSE OF EIF2AK1 HRI TO HEME DEFICIENCY | -1.084342137 | -0.034249446 | -10.57189537 | 2.72E-16 | 7.85E-13 | 26.62277542 |
| WP NEUROINFLAMMATION | -1.043950399 | -0.044491397 | -9.536861566 | 2.14E-14 | 1.97E-11 | 22.40485891 |
| REACTOME REGULATION OF GENE EXPRESSION BY HYPOXIA INDUCIBLE FACTOR | -1.035862136 | -0.037366336 | -9.581908902 | 1.77E-14 | 1.97E-11 | 22.59023274 |
| WP PATHOPHYSIOLOGICAL ROLES OF DUX4 IN FSHD1 | -0.95216362 | -0.024150158 | -8.850132045 | 4.02E-13 | 1.93E-10 | 19.56419239 |
| WP OXIDATIVE PHOSPHORYLATION | 0.900691158 | 0.0150493 | 7.261568013 | 3.66E-10 | 3.92E-08 | 12.96183154 |
| KEGG MEDICUS VARIANT MUTATION INACTIVATED PINK1 TO ELECTRON TRANSFER IN COMPLEX I | 0.870938825 | 0.020305813 | 6.53244848 | 7.99E-09 | 3.98E-07 | 9.976064162 |
| PID HIF2PATHWAY | -0.859154004 | -0.041837069 | -9.479725064 | 2.73E-14 | 1.97E-11 | 22.16953987 |
| KEGG MEDICUS REFERENCE ANTIGEN PROCESSING AND PRESENTATION BY MHC CLASS II MOLECULES | 0.858885522 | 0.033286588 | 4.867610232 | 6.48E-06 | 7.92E-05 | 3.528424343 |
| REACTOME COMPLEX I BIOGENESIS | 0.858450777 | 0.022827993 | 6.751548711 | 3.18E-09 | 1.95E-07 | 10.86693856 |
| KEGG MEDICUS REFERENCE ELECTRON TRANSFER IN COMPLEX I | 0.852965514 | 0.012852842 | 6.214752217 | 3.00E-08 | 1.02E-06 | 8.69710186 |
| WP MITOCHONDRIAL COMPLEX I ASSEMBLY MODEL OXPHOS SYSTEM | 0.852204754 | 0.021936735 | 6.610321392 | 5.77E-09 | 3.14E-07 | 10.29195339 |
| KEGG MEDICUS VARIANT MUTATION CAUSED ABERRANT SNCA TO ELECTRON TRANSFER IN COMPLEX I | 0.848635118 | 0.01511806 | 6.241884762 | 2.68E-08 | 9.55E-07 | 8.805663072 |
| WP SREBF AND MIR33 IN CHOLESTEROL AND LIPID HOMEOSTASIS | -0.847297039 | -0.018749196 | -7.665326084 | 6.51E-11 | 1.25E-08 | 14.63461879 |
| KEGG MEDICUS PATHOGEN HTLV 1 TAX TO NFY MEDIATED TRANSCRIPTION | 0.845860111 | 0.023259414 | 5.282219952 | 1.30E-06 | 2.17E-05 | 5.06459573 |
| BIOCARTA NPC PATHWAY | -0.843358174 | -0.067907382 | -5.756826623 | 1.96E-07 | 4.52E-06 | 6.887110097 |
| REACTOME HDMS DEMETHYLATE HISTONES | -0.833574074 | 0.012247928 | -7.296978605 | 3.15E-10 | 3.92E-08 | 13.1081376 |
| WP MAPK PATHWAY IN CONGENITAL THYROID CANCER | -0.826100601 | -0.001067262 | -7.081007927 | 7.89E-10 | 6.32E-08 | 12.21734279 |
| KEGG MEDICUS REFERENCE GPI ANCHOR BIOSYNTHESIS | 0.82579598 | 0.009217546 | 7.955325111 | 1.88E-11 | 4.92E-09 | 15.8404908 |
| KEGG MEDICUS PATHOGEN HPV E6 TO NOTCH SIGNALING PATHWAY N00380 | -0.820469682 | 0.022126069 | -5.939731361 | 9.31E-08 | 2.46E-06 | 7.604708264 |
| WP BMP2 WNT4 FOXO1 PATHWAY IN PRIMARY ENDOMETRIAL STROMAL CELL DIFFERENTIATION | -0.816152277 | -0.014549713 | -7.278640351 | 3.40E-10 | 3.92E-08 | 13.032358 |

GSVA，Gene Set Variation Analysis.
